# Fourier synthesis optical diffraction tomography for kilohertz rate volumetric imaging

**DOI:** 10.1126/sciadv.adr8004

**Published:** 2025-08-13

**Authors:** Peter T. Brown, Nikta Jabbarzadeh, Luis Meneses, Kaia Swanson, Aidan Pintuff, Ekaterina Monakhova, Rory Kruithoff, Navish Wadhwa, Domenico F. Galati, Douglas P. Shepherd

**Affiliations:** ^1^ Center for Biological Physics, Arizona State University, Tempe, AZ 85287, USA.; ^2^ Department of Physics, Arizona State University, Tempe, AZ 85287, USA.; ^3^ Biodesign Center for Mechanisms of Evolution, Arizona State University, Tempe, AZ 85287, USA.; ^4^ Biology Department, Western Washington University, Bellingham, WA 98225, USA.

## Abstract

Many biological and soft matter processes occur at high speeds in complex three-dimensional (3D) environments, and developing imaging techniques capable of elucidating their dynamics is an outstanding challenge. Here, we introduce Fourier synthesis optical diffraction tomography (FS-ODT), a quantitative phase imaging approach capable of recording the 3D refractive index at kilohertz rates. FS-ODT introduces computational strategies that multiplex tens of illumination angles in a single tomogram, markedly increasing the volumetric imaging rate. We validate FS-ODT on samples of known composition, hindered diffusion of colloids in solution, and the motility of bacterial swimmers. We also integrate FS-ODT into a multimodal microscope combining refractive index imaging with multicolor structured illumination microscopy. FS-ODT is a promising approach for unlocking imaging regimes that have been little explored, including understanding the physical interactions of colloids and microswimmers with their 3D environment and the interplay between these stimuli and the molecular response of biological systems.

## Teaser

Highly multiplexed diffraction tomography enables kilohertz volume imaging for dynamic soft matter and biological systems.

## INTRODUCTION

Probing biology and physics at high resolution and speed in three dimensions is a tremendous experimental challenge that has inspired notable advances in microscopy techniques across time and length scales. Fluorescence imaging is one of the most widely used techniques, with many existing variants addressing different experimental needs from the nano- to macroscale. Fundamentally, all fluorescence methods are subject to the “triangle of frustration,” where photobleaching, phototoxicity, and fluorophore photophysics limit the speed, duration, and total light dose (*1*).

Recent advances in quantitative phase imaging (QPI) techniques have positioned these as an alternative to fluorescence in many domains (*2*). In contrast to fluorescence microscopy, image contrast in QPI originates from the sample’s refractive index (RI), avoiding the aforementioned low-signal levels, photobleaching, and the triangle of frustration associated with fluorescent labeling. When morphology and dynamics are of primary interest, QPI approaches are becoming a method of choice for long-term, volumetric imaging at few-hertz rates (*3*).

For acquisition rates beyond a few hertz, a variety of single-shot QPI approaches enable fast “volumetric” imaging, including digital in-line holography (*4*), off-axis holography (*5*), and interferometric scattering microscopy (iSCAT) (*6*) (Table 1). However, single-shot QPI methods can only obtain unambiguous volumetric reconstructions by putting strong priors on the geometry of particles that are imaged, e.g., spheres, rods, or other particles with a known scattering model. Such approaches commonly fail in dense samples and have limited axial resolution (*7*).

**Table 1.** Comparison of kilohertz-scale volumetric imaging methods. RI, F, and SHG stand for refractive index, fluorescence, and second-harmonic generation, respectively. The QPI techniques are discussed in the text. SLIM and eventLFM are light-field microscopy techniques.

Method	Speed	Limit	Contrast	True 3D	Ref.
Digital holographic microscopy	5 MHz	Camera	RI	No	(*71*)
iSCAT	20 kHz	Camera	RI	No	(*58*)
SLIM	1 kHz	Camera/multiplexing	F	Yes	(*72*)
eventLFM	1 kHz	Sample/accumulation time	F	Yes	(*73*)
Holographic SHG	1.5 kHz	Camera	SHG	No	(*74*)
ODT	0.1 kHz	DMD/camera	RI	Yes	See Introduction
FS-ODT	1 kHz	DMD/multiplexing	RI	Yes	This work

In contrast, optical diffraction tomography (ODT) (*8*–*12*), infers the three-dimensional (3D) RI of a sample by combining multiple views obtained by projecting coherent light through the sample at different angles. At a high level, ODT is closely related to synthetic aperture microscopy (*13*) and Fourier ptychography (*14*). ODT distinguishes itself from other “volumetric” QPI approaches in its flexibility to probe arbitrary refractive indices and nonsparse samples and obtain true volumetric images. Therefore, ODT stands out as the method of choice for imaging dense samples in complex environments, as evidenced by a number of recent biological applications including measuring cell dry mass (*15*), flow cytometry (*16*), traction force microscopy (*17*), and 3D histopathology (*18*).

Classically, ODT approaches have collected ≳100 views to perform high-quality tomographic reconstruction. For such a large number of individual views, ODT imaging is typically two orders of magnitude slower than single-shot techniques. In reality, there exists a trade-off between the number of ODT views, desired reconstruction quality, and achievable imaging speed. For example, prior works demonstrated high-quality tomographic reconstruction from limited views (*19*) and inference of missing information from limited views using supervised deep learning (*16*). Ultimately, the primary hurdle to improve ODT acquisition speed remains the rate of pattern generation for individual ODT views. Achieving high-quality RI reconstructions at kilohertz volumetric imaging speeds across diverse sample types is still an open challenge. Commonly, the angle that the illumination traverses the sample is controlled by galvanometric mirrors (*20*) or spatial light modulators (SLMs) (*21*–*25*). The fastest and most common SLM choice is the digital micromirror device (DMD), a binary device often run in a time-multiplexed grayscale mode that limits the pattern refresh rate to 1 kHz (*26*, *27*). DMDs are capable of pattern changes at 10 kHz in binary mode, but this introduces stray diffraction orders that must be filtered out using static (*22*) or dynamic (*16*, *28*) masks. Even working at the full DMD display rate, achieving high-quality ODT using >100 patterns limits the volumetric frame rate to <100 Hz, still short of the volumetric kilohertz target.

Previous approaches to accelerating diffraction tomography have relied on multiplexing wavelengths, which has the advantage that different views are incoherent, but requires engineering inflexible custom optics. Furthermore, these approaches have not been demonstrated at high speeds or on dynamic samples (*29**–**31*).

To address the challenge of realizing high-resolution, true-3D, kilohertz-rate, and label-free volumetric imaging, we developed an alternative strategy to rationally designing and projecting ODT illumination patterns, Fourier synthesis optical diffraction tomography (FS-ODT). FS-ODT builds on our previous DMD pattern generation tools (*32*) to enable multiplexing many ODT views into a single image. We refer to this strategy as Fourier synthesis because we construct the complex illuminations patterns required for multiplexing by combining simple atomic patterns in the Fourier plane. Because FS-ODT places the DMD in a conjugate Fourier plane to the objective and achieves position and angle control over the patterns using a spatial carrier frequency, it is possible to increase the information content in a single image orders of magnitude further than multiplexing Lee holograms (*16*). Although multiplexed intensity diffraction tomography (IDT) also synthesizes complex patterns using multiple light-emitting diode point sources in the far field (*33*–*35*), FS-ODT's use of a fast pattern modulator enables additional control over the beam phase and position and allows us to achieve two orders of magnitude faster volume acquisition.

Increasing the information content per image by multiplexing introduces a more ill-posed reconstruction problem. To address the ill-posed problem of multiplexed patterns, we designed an iterative reconstruction approach based on multislice beam propagation and accelerated proximal gradient descent. We additionally created a “demultiplexing” approach to generate a high-quality initial 3D RI distribution and created approaches to avoid nonoptimal solutions due to phase wrapping.

We demonstrate the capabilities of FS-ODT for a variety of samples and scenarios, illustrating its unique combination of high-quality RI reconstruction and high-speed volumetric imaging capability. We first profile the reconstruction quality versus the degree of angle multiplexing by imaging a sample of known composition, a 10-μm polystyrene microsphere (PMS) and a biological sample of known morphology, *Tetrahymena*. Next, we consider dynamic samples, including diffusing PMS and motile bacteria, and demonstrate FS-ODT tracking of colloidal particles and extraction of their hydrodynamic properties. Last, we combine position and angle multiplexing at the fastest FS-ODT imaging rate possible with our hardware to measure diffusing microspheres at kilohertz volumetric frame rates. In addition to QPI measurements, we further demonstrate multimodal microscopy, combining multicolor 2D structured illumination microscopy (2D-SIM) and FS-ODT on *Tetrahymena* samples. Together, the results presented here demonstrate that FS-ODT is a powerful approach for the dynamics of cells and colloids in complex environments.

## RESULTS

### Fourier synthesis of ODT patterns

**Fig. 1.**
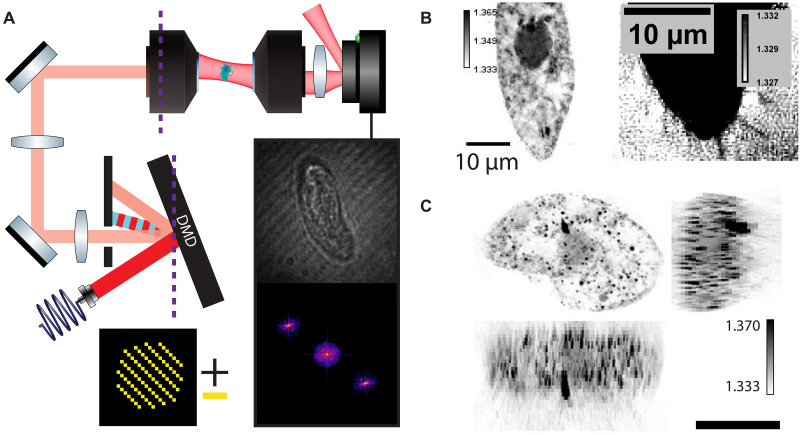
Fourier synthesis optical diffraction tomography. (**A**) In FS-ODT, a spot-like pattern with a superimposed spatial carrier frequency is displayed on the DMD (bottom left), which is conjugate to the back-focal plane of the objectives (purple line). The light that diffracts from the − mirrors (red) at the carrier frequency is transmitted, whereas the light that diffracts from the + mirrors (blue) is blocked. The position and carrier frequency of the spot pattern control the angle and position of the ODT illumination, respectively. (**B**) Fixed *Tetrahymena* imaged with nonmultiplexed FS-ODT shown in a single axial plane. Internal cell structures are resolved (left), and 200-nm cilia are at the edge of our detection ability (right). (**C**) MIPs of the *Tetrahymena* RI in the *xy* (top left), *xz* (bottom left), and *zy* planes (right). The internal structure of the cell can be resolved, including the nucleus and the OA. The unusual morphology suggests that this cell may be in the process of dividing. Scale bar, 20 μm.

In FS-ODT, we generate patterns using a DMD conjugate to the objective back focal (or Fourier) plane, as shown in Fig. 1A. Plane waves are encoded by circular “spot” patterns on the DMD. Varying the beam position in the sample corresponds to a adding a phase ramp in the Fourier plane, and so to make the beam position tunable, we align the system so that beams that diffract from a given spatial carrier frequency on the DMD are centered in the optical system. Beam angle through the sample is encoded by spot position, so multiplexing tens to hundreds of beams at different angles is possible by displaying multiple spatially separated spot patterns on the DMD. Furthermore, by working in the Fourier plane we avoid the need to filter stray diffraction orders as these are mitigated by Fourier broadening due to the small spot sizes. We have carefully modeled this approach by extending the DMD simulation tools we developed previously for multicolor SIM (*32*). For a more detailed description of the experimental setup, see section S2.

To generate a single plane wave at spatial frequency $f=\left(f_{x},f_{y}\right)$ displaced from the center of the field of view by δr, we generate a spot pattern at location $r_{p}$ and spatial frequency $f_{p}$ on the DMD. The object space parameters are determined by

$$\delta r=\frac{f_{l}\lambda }{M}\left(f_{p}-f_{c}\right)$$ (1)

$$f=\frac{M}{f_{l}\lambda }\left(r_{p}-r_{c}\right)$$ (2)

where $f_{c}$ is the spatial carrier frequency, $r_{c}$ is the position on the DMD aligned with the optical axis, $f_{l}$ is the focal length of the objective lens, *M* is the magnification between the DMD and the lens back focal plane, and λ is the wavelength of light. Because of the Fourier transform relationship between the DMD and illumination patterns, smaller DMD spot patterns generate larger image-space patterns. In our setup, 40-mW incident on the DMD yields 2 μW entering the microscope objective for a single ODT plane wave. Nevertheless, because ODT detects transmitted light, there is sufficient signal to image with <100-μs exposure times (section S2A). The trade-off inherent to placing the DMD in the Fourier plane could be mitigated by either displacing the DMD out of the Fourier plane and accounting for the resulting defocus using a Lee hologram–type approach or by adopting the alternative optical configuration demonstrated in (*36*).

To validate that the proposed FS-ODT optical design is capable of high-quality 3D RI reconstruction, we first performed nonmultiplexed ODT on a complex 3D sample, fixed *Tetrahymena* cells (Fig. 1, B and C). After RI reconstruction, we resolve the internal structure of the cell, including the nucleus, the oral apparatus (OA), and the cilia. The cilia are expected to be 0.2 μm in diameter, which is much smaller than the Abbe limit for our detection optics, 2NA/λ ∼ 0.39 μm (where NA is the numerical aperture). As such, the measured RI contrast is small due to averaging of the structure and background over the resolution voxel size. Reconstruction parameters are provided in table S1.

We infer the RI distribution using custom GPU-accelerated Python code, which implements accelerated proximal gradient descent using the fast iterative shrinkage-thresholding algorithm (FISTA) (*37*) (Materials and Methods and section S3). We include the physics of light propagation through the RI using either the beam-propagation model (BPM) or the split-step non-paraxial (SSNP) forward models (*35*, *38*). FISTA and related proximal approaches are particularly powerful for solving ill-posed inverse image reconstruction problems because they provide a framework for applying regularization. Regularization stabilizes the reconstruction and favors physical solutions from all possible RI distributions consistent with the data. We use total variation regularization, which promotes smoothness, and $\ell_{1}$ regularization, which promotes sparsity. In addition, we impose physical constraints, typically that the imaginary part of the RI is strictly absorptive and the real part of the RI is greater than the background index.

**Fig. 2.**
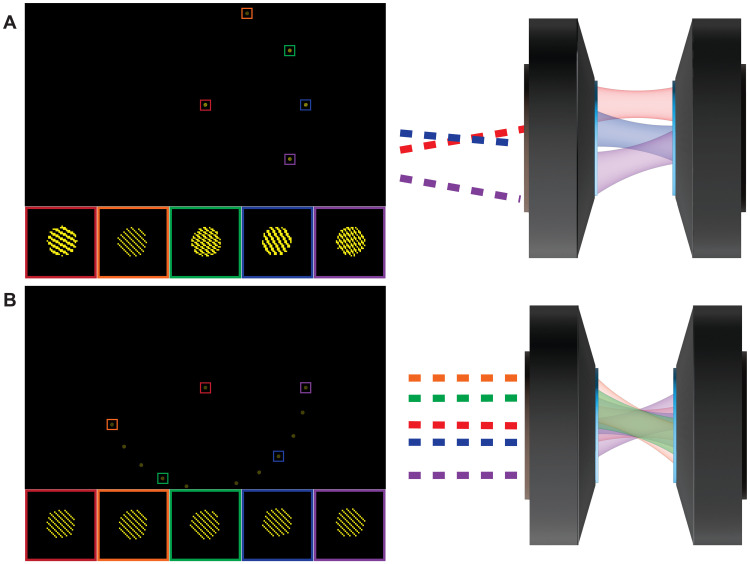
Multiplexed illumination with FS-ODT. (**A**) Exemplary position multiplexing pattern. Five spot patterns are displayed across the DMD face (top left) with different carrier frequencies (inset, bottom left). When relayed to the objective back focal plane (right), the resulting beams have different incidence angles, and the lens transforms these angles to different positions in the focal plane. Only three beams are shown for clarity. (**B**) Exemplary angle multiplexing pattern. Twelve spot patterns (top left) are displayed at different spatial positions but with the same carrier frequency. Five selected patterns are shown in more detail (inset, bottom left). When relayed to the objective back focal plane (right), the resulting beams have different incidence positions, and the lens transforms these positions to different angles in the focal plane. Only five beams are shown for clarity.

Next, we apply the FS-ODT pattern generation strategy to generate multiplexed illumination patterns. Because FS-ODT uses spatially separated spot patterns to generate plane waves at different angles, we generate multiplexed patterns by displaying spot patterns at different positions on the DMD. We synthesize composite patterns by specifying the beam frequency, position shift, phase, and diameter for each spot pattern $\left\{(f_{1},\delta r_{1},\phi_{1},d_{1}),\ldots \left(f_{N},\delta r_{N},\phi_{N},d_{N}\right)\right\}$. Each pattern generates *N* beams passing through the sample plane in different positions at different angles (i.e., spatial frequencies). Unlike in multiplexed IDT approaches, these beams are mutually coherent, and so their combination generates a complex illumination pattern at the sample. Our approach enables two forms of multiplexing: (i) position multiplexing to extend the system field of view and (ii) angle multiplexing, which illuminates a single sample region with overlapping beams at different angles. We demonstrate these two strategies in Fig. 2. A unique benefit of FS-ODT is that these two strategies can be combined to enable angle multiplexing over a large field of view.

As illustrated in Fig. 2, changing the spot carrier frequencies or positions changes the alignment of the spots with the DMD pixel grid. Although altering the active mirrors may affect the relative power in the different ODT plane waves, the effect is negligible because the power is related to the spatial Fourier component at the carrier frequency. The number of active mirrors controls the dc Fourier component, but FS-ODT does not use the beam diffracted by the dc component. Our reconstruction approach accounts for any remaining slight power variation between different ODT plane waves.

In this work, we typically use spot patterns with a diameter of *d_i_* = 20 mirrors, resulting in imaging space patterns with a diameter of 30 μm. Although it would be advantageous to have larger imaging space beams, speckle and diffraction from DMD imperfections corrupt our imaging patterns when working with smaller DMD spot patterns. Instead, we mitigate the disadvantage of relatively small illumination beams through FS-ODT position multiplexing. To image a 100 μm–by–100 μm field of view for a typical high-NA objective lens, it is possible to either scan sequentially using 15 pattern sets with no position multiplexing or perform a single-shot using 15× position multiplexing that covers the entire field of view.

### Low-resolution Rytov demultiplexing

Iterative ODT reconstruction, as described above, relies on a high-quality initial guess for the RI distribution. Because the problem is not globally convex, the iteration scheme may not converge if the guess is too far from the true RI distribution. The initial guess is most commonly generated from linear weak scattering approximations, such as the Born and Rytov approximations, that can be efficiently computed from the electric field data. For plane wave illumination, these approximations relate the 2D Fourier transform of the image electric field to a spherical cap in the 3D Fourier transform of the scattering potential (section S3).

Multiplexing many patterns results in a more ill-posed inverse problem than single-beam ODT due to the additional need to unmix scattering contributions from different incident plane waves. When the illumination pattern contains multiple plane waves, it is not obvious which spherical cap each image Fourier frequency should be assigned. This ambiguity can be removed experimentally using phase shifting, but this requires additional images and negates the speed advantage of multiplexing.

**Fig. 3.**
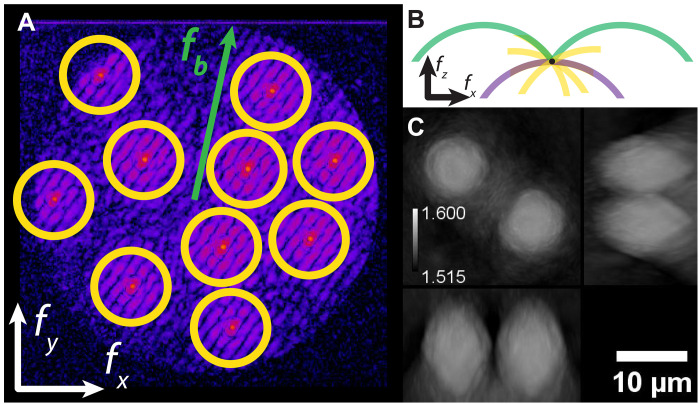
Low-resolution Rytov demultiplexing. (**A**) In the Fourier transform of each off-axis hologram, we select regions around each plane wave frequency where the scattered light is dominated by that individual plane wave (yellow circle). The radius of these regions is much smaller than the bandpass frequency of the microscope, $f_{b}$, so these regions contain low-resolution information about the RI object. (**B**) The information from each low-resolution region is mapped to the correct position (yellow arcs) in the 3D Fourier space representation of the scattering potential. These regions are centered about zero frequency (black dot). For reference, we show arcs corresponding to a straight-on incident beam (purple) and beams at the band pass frequency (green). (**C**) We obtain an initial guess for the RI after inverse Fourier transforming the scattering potential. For the 10-μm-diameter PMS shown here, the low-resolution demultiplexed guess in the Rytov approximation provides an excellent starting point for further optimization.

To improve the convergence of our reconstruction algorithm, we develop an approach to initialize the RI with a high-quality guess in the presence of multiplexing, which we refer to as low-resolution Rytov demultiplexing. In this scheme, we observe that, in many common experimental situations, information from the scattering of the *i*th plane wave dominates the hologram in the region around the plane wave carrier frequency. We expect this situation to prevail if scattering is weak enough and, for example, the sample RI is greater than the background and its power spectrum decays with increasing spatial frequency. For each plane wave, we select the region in Fourier space that is closer to that carrier frequency than to any other. We take the resulting *N* lower-resolution demultiplexed fields and compute the corresponding Rytov phases (eq. S16). Last, we generate an initial RI distribution by using the linear scattering model to map this information to the scattering potential at the correct position in Fourier space. An example of this process is shown in Fig. 3 (A and B) for 10× multiplexing for a sample consisting of a 10-μm-diameter PMS. The resulting Rytov demultiplexed RI (Fig. 3C) captures the features of the sample and is a good initial guess for the solver.

Although we find Rytov demultiplexing produces a better initial guess than, e.g., beginning with a constant RI, it eventually fails because as the degree of multiplexing increases, the resolution of the demultiplexed images decreases. For small objects and high degrees of multiplexing, the demultiplexed images do not contain sufficient detail to accurately represent the RI. This problem is particularly acute for thick objects that produce phase-wrapped electric fields because the phase wraps must be detected by comparing phase changes across the object. When rapid phase variations are not captured by the low-resolution demultiplexed images, phase unwrapping breaks down and phase errors in the initial guess can lead to rapid deterioration of the RI reconstruction.

In some cases, the sensitivity of the optimization problem to accurate phase unwrapping can be mitigated by using a loss function, which includes a phase-sensitive and a phase-insensitive term (eq. S5). This illustrates that, although the phase information obtained by ODT produces a less ill-poised reconstruction problem, it simultaneously produces a more complex landscape for the loss function, which poses a difficult challenge for convex optimization approaches.

### FS-ODT validation

As an initial validation of our approach, we collect FS-ODT images of a sample of known structure and RI, a 10-μm-diameter PMS suspended in immersion oil, using a range of different multiplexing conditions. We consider multiplexing by factor of 1, 3, 10, and 19 using a fixed set of *N* = 147 plane wave directions. Where the total number of angles is not divisible by the multiplexing factor, we include some plane waves twice to simplify the reconstruction.

Although FS-ODT can generate arbitrary multiplexed patterns, reconstruction quality is improved using multiplexed patterns that are easier to unmix by ensuring the beam frequencies in each image are as far apart as possible. This maximizes the resolution achieved in the Rytov demultiplexing initialization. We design our patterns using an iterative algorithm to ensure this is the case. We initialize each *M*-fold multiplexed pattern with *M* beam angles by iteratively selecting the beam angle that maximizes the distance from those already chosen. Next, we iterate over all choices of two patterns and randomly swap beam angles. We define a loss function, the average distance between beam angles on the DMD up to a certain maximum value. If swapping the angles increases the loss function, we keep the swap. We obtain high-quality multiplexed patterns after performing five iterations of 300 swaps.

Because of the thickness and large RI contrast between the PMS and the immersion oil, $n_{\text{PMS}}∼1.57$ and $n_{o}∼1.515$, we use the SSNP for our reconstruction (*38*) to both allow for multiple scattering and accurately model the propagation of the complex illumination beam. The BPM with an obliquity factor correction achieves similar performance with reduced memory and computational requirements for single plane wave illumination (*35*), but in our case, the multiple incident plane waves do not share a common obliquity factor, so the SSNP is more appropriate.

**Fig. 4.**
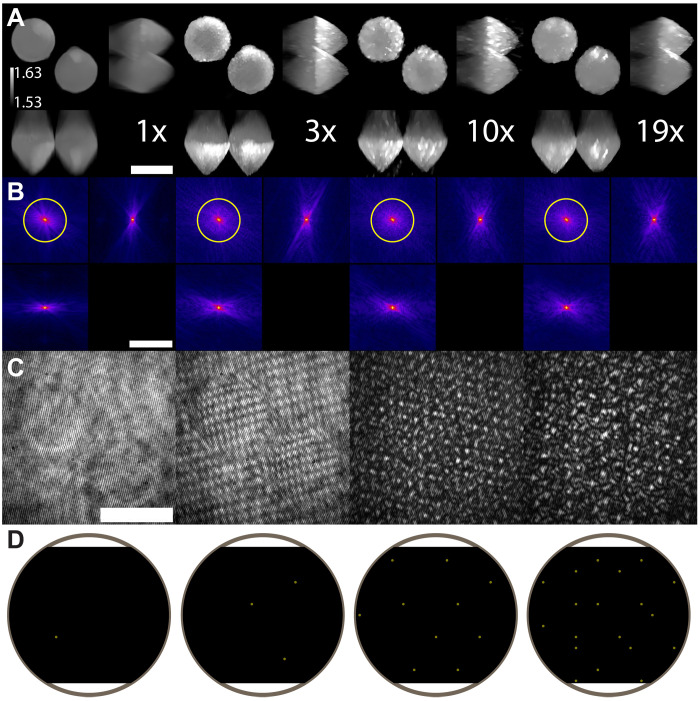
10-μm microsphere RI reconstruction versus angle multiplexing. (**A**) RI reconstruction using the SSNP and an initial guess from the low-resolution demultiplexed Rytov approach described in the text. MIPs through the microsphere are shown in the *xy* plane (top left), *yz* plane (top right), and *xz* plane (bottom left). Results for angle multiplexing factors of 1, 3, 10, and 19 are shown (left to right). Scale bar, 10 μm. (**B**) Magnitude of the 3D Fourier transforms of the scattering potentials corresponding to the RI reconstructions shown in (A). The orthogonal images shows slices along the $f_{z}=0$ (top left), $f_{y}=0$ (bottom left), and $f_{x}=0$ (top right) planes. Magnitudes are displayed using a gamma of 0.25. The maximum recoverable spatial frequency information in the weak-scattering approximations is illustrated in yellow. Scale bar, 5 μm^−1^. (**C**) Exemplary raw hologram images, which are used to recover the bead RI. Scale bar, 10 μm. For higher degrees of multiplexing, these become more complex due to the mutual interference of many plane waves. (**D**) Exemplary DMD patterns used to generate the multiplexed illumination patterns in (B). Only the portion of the DMD within the detection objective pupil radius (gray circle) is shown. The DMD is slightly smaller (~84%) than the pupil along the vertical direction.

We find that FS-ODT achieves high-quality 3D RI reconstructions of the PMS for 1× (no multiplexing), 3×, 10×, and 19× multiplexing (Fig. 4). With no multiplexing, we recover a nearly spherical object of the correct diameter with *n* ≈ 1.57, as expected because this problem is the least ill-posed and has the largest effective signal-to-noise ratio (SNR) per beam. Because all raw images are acquired with similar peak intensity, the effective SNR per beam is reduced as the degree of multiplexing increases. As expected, because of the missing cone problem, the sphere appears somewhat elongated in the axial direction. The 3×, 10×, and 19× multiplexed data also produce high-quality reconstructions, but the RI is not distributed as uniformly along the optical axis. This reconstruction artifact is reminiscent of missing-cone effects and could be a result of effective loss of SNR for individual plane waves in the highly multiplexed beam.

We numerically explore and further validate our multiplexed FS-ODT approach by reconstructing synthetic images generated using custom GPU accelerated Mie scattering code (section S1). We find that multiplexed patterns enhance the RI reconstruction compared with nonmultiplexed patterns when the number of raw tomograms is kept fixed.

### Hindered diffusion and microswimmer motility

Having established that FS-ODT produces high-quality RI reconstructions in static samples, we now apply our approach to study hindered diffusion, hydrodynamic interactions, and microswimmer motility. Phase imaging approaches offer advantages over fluorescence imaging in lower phototoxicity, faster frame rates, and axial position sensitivity. As such, phase methods are ideally suited to studying motion in 3D environments, such as the motility of colloids or cells in complex environments. One poorly understood question is how cells swim in viscoelastic environments created by the presence of inert colloids or polymers in solution (*39*). This topic is of broad interest because these fluids mimic the natural environment of bacterial species better than typical in vitro experiments and may provide insight into behavior and evolutionary pathways, which is currently lacking. Complex environments may also affect collective bacterial dynamics, including swarming and biofilm formation, which are interesting from a fundamental perspective of better understanding active matter as well as from a public health perspective (*40*). 3D QPI approaches offer advantages over 2D-only or 2D with limited *z*-range tracking, which only measure particles at or near the microscope’s focal plane, particularly in dense environments like bacterial suspensions in complex fluids. As a first step toward addressing these questions, we demonstrate that we can image and distinguish diffusing microspheres and swimming *Escherichia coli* cells in 3D using FS-ODT.

Imaging dense samples introduces new reconstruction challenges, as our algorithms require knowledge of the excitation electric field with no sample present to distinguish electric field patterns generated by the complex illumination pattern from those due to interaction with RI objects. One standard solution is to acquire a background image taken at a spatial position where no RI objects are present. However, for dense diffusing objects, there are no sample-free regions. Instead, we rely on the time-average image, assuming that, over the long term, the RI inhomogeneities average out. For relatively sparse samples, this is a good approximation. For denser samples, it may be necessary to account for an average background RI different from the fluid. Other approaches, including joint inference of the incident electric field and the sample RI, are possible (*41*).

**Fig. 5.**
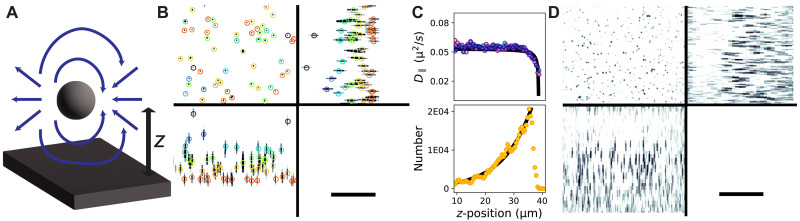
Hindered diffusion of colloidal microspheres. (**A**) Colloidal particles interact with nearby boundaries when the boundary influences the fluid flow fields generated by the particles’ motion. (**B**) A 1-μm-diameter PMS diffusing in a water-glycerol mixture imaged with FS-ODT. MIPs of the RI are shown in three orthogonal planes. Circles indicate the bead location determined by tracking algorithm. Color represents the axial position from near the coverslip (red) to up in the sample (purple). Scale bar, 20 μm. (**C**) Hydrodynamic interactions between microspheres and the coverslip induce a spatially varying diffusion coefficient (top). The microspheres are buoyant in this solution, leading to a spatially varying density profile (bottom). (**D**) FS-ODT resolves individual microspheres in a solution with 10× greater PMS concentration compared with (B). Scale bar, 20 μm.

We applied FS-ODT to study hindered diffusion of 1-μm beads in a water-glycerol mixture and quantified the bead dynamics by determining the diffusion coefficient as a function of distance to the coverslip using 3D tracking (Fig. 5 and movies S1 and S2). The diffusion coefficient is sensitive to hydrodynamic forces on the beads and serves as an indicator of hydrodynamic interactions. Colloidal particles experience hydrodynamic interactions with boundaries because they generate fluid flow fields as they move, which must satisfy the Stokes equation with appropriate boundary conditions. When a sphere is within a few radii, *R*, of a boundary, the no-slip boundary condition at the wall modifies the flow fields (Fig. 5A), which affects the mobility of the sphere and therefore the translational and rotational diffusion coefficients. This effect, known as hindered diffusion, is perturbative in the ratio of the sphere’s radius to the distance from the wall R/h. To leading order, the diffusion coefficient parallel to the wall is (*42*)

$$D_{∥}=D_{o}\left[1-\frac{9}{16}\frac{R}{h}+O\left(\frac{R^{2}}{h^{2}}\right)\right]$$ (3)

where $D_{o}$ is the diffusion coefficient in a unbounded fluid. Our measured diffusion coefficients versus height above the coverslip (Fig. 5B) match well this functional form. There are small deviations from the expected curvature, which we attribute to the fact that the microspheres sample different heights during our measurement, leading to some broadening of the measured curve.

Hindered diffusion has previously been studied using evanescent wave dynamical light scattering (*43*) and optical tweezer experiments combined with in-line holography (*44*). FS-ODT provides an alternative approach that can resolve hydrodynamic forces over tens of micrometers axially without the need to perturb the sample, scan tweezer positions, or combine multiple imaging techniques. ODT’s unique ability to image across a 3D field of view and in multiple scattering environments enables simultaneously tracking of many particles in dense colloidal suspensions, which is extremely challenging using these other approaches.

**Fig. 6.**
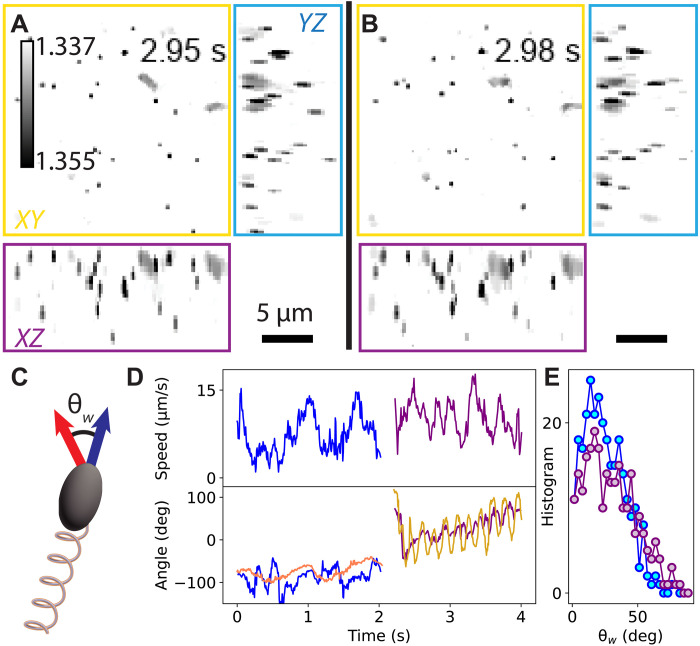
*E. coli* motility. (**A**) RI reconstruction of bacteria and tracer particles MIP along three orthogonal axes, *XY* (top left), *YZ* (right), and *XZ* (bottom). The bacteria and tracers can be distinguished based on their RI and morphology. Two bacteria (top right) and ~20 tracer particles are visible in the *XY* projection. (**B**) Bacteria and tracer particles 30 ms later. One bacterium has rotated, and the tracers have changed position due to diffusion and advection. (**C**) During bacterial swimming, we define the body axis (blue) and the average velocity (red). The wobble angle, θ_*w*_, is the average angle between the two. (**D**) Speed (top) and 2D orientation of the bacteria axis (red and gold) and the bacteria velocity (blue and purple). Different traces represent the two swimming bacteria observed in the experiment. deg, degrees. (**E**) Histogram of measured wobble angles. Colors correspond to bacteria identified in (D).

Next, we studied *E. coli* motility (*25*, *45*) by imaging bacteria in solutions seeded with 0.5-μm PMS tracer particles at volumetric rates of 143 Hz over ~7 s (Fig. 6 and movies S3 and S4). We found that the bacteria and tracer particles can be distinguished based on RI and morphology. We observe diffusion of the tracer particles and directed swimming of two *E. coli* cells in this dataset. To quantify the swimming behavior of the *E. coli*, we trained a classifier to segment the cell bodies and then tracked their position and orientation (Fig. 6D). From these 4D data, we computed the wobble angle, which is the average angle between the cell’s average velocity and body axis (Fig. 6C). Wobble angles have previously been used to characterize *E. coli* swimming in complex fluids (*39*). This experiment demonstrates that FS-ODT can effectively identify bacteria swimming in 3D and be used to compute wobble angles, which we anticipate will enable larger-scale studies.

### Diffusing microspheres imaged at kilohertz volumetric rates

**Fig. 7.**
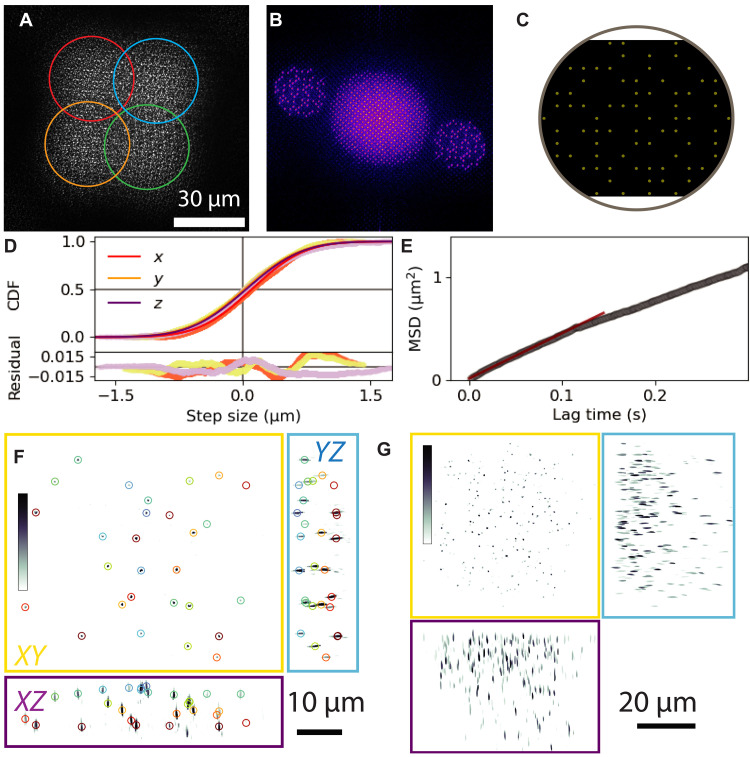
Diffusing microspheres at kilohertz volumetric rates with an extended field of view. (**A**) Intensity profile of the beam after interacting with microspheres using 19× angle multiplexing and 4× position multiplexing (circles). Position multiplexing expands the usable field of view by ∼2×. (**B**) Fourier transform of the intensity profile in (A) showing 76 distinct beams in Fourier space. (**C**) DMD pattern used to generate the illumination shown in (A) and (B). (**D**) Step-size CDF (points), Gaussian model fits (lines), and residuals (bottom) for steps separated by 200 frames along the *x* (red-orange), *y* (yellow), and *z* (purple) directions. (**E**) Ensemble-average MSD versus lag time (gray) and a linear fit (red) up to a lag time of 0.144 s. (**F**) RI reconstruction at a single time point for a 10-μm tall chamber. RI values are shown between 1.352 and 1.413. PMSs are localized (circles) and tracked. (**G**) RI reconstruction at a single time point for a denser sample in a 120-μm tall chamber. The RI scale is the same as in (F).

We next demonstrate that FS-ODT can reliably image diffusing colloidal particles at kilohertz volumetric rates. Specifically, we image 1-μm PMS diffusing in water over a period of 0.969 s at a volumetric rate of 1.032 kHz with a field of view of 20.5 μm (*z*) by 96 μm (*y*) by 104 μm (*x*) (movies S5 and S6). At each time point, we collect eight images using 19× angle multiplexed patterns. In addition, we expand the field of view of the imaging system by expanding the illumination pattern 2× in each direction by including spots with four different carrier frequencies. This combined angle and position multiplexing uses a total of 608 plane wave patterns. We show an exemplary angle- and position-multiplexed illumination pattern in Fig. 7 (A to C).

We performed RI reconstruction at each time point (Fig. 7F) and tracked the diffusing PMS to extract their diffusion coefficients. As for other time-resolved experiments, we construct a reference electric field by time-averaging the electric fields obtained from off-axis holography. We quantify the diffusive motion by calculating cumulative distribution functions (CDFs) of the step-size distribution along each axis (Fig. 7D) and the mean square displacement (MSD) of the beads versus lag time (Fig. 7E). For short lag times, the CDFs can be strongly affected by localization error. Therefore, we examine the CDFs for a lag time of 0.194 s (200 frames) and find that the CDFs closely match the expected Gaussian form. We extract the ensemble-average diffusion coefficient by performing a linear fit to the MSD and determine $D=0.68\;{\mathrm{\mu m}}^{2}\;s^{-1}$.

We display volumetric data in Fig. 7 using maximum intensity projections (MIPs) along three orthogonal axes. We can easily resolve the diffusing PMS, and we find that they are distributed over an axial range of about 10 μm, which is the approximate height of the sample chamber (Fig. 7F). We also imaged the PMS diffusing in a 120-μm-thick chamber, focusing on the region within 40 μm of the coverslip (Fig. 7G). These images provide an ideal starting point for particle tracking, particle imaging velocimetry, or other approaches to either explore the physics of colloidal suspensions or use these colloids as tracer particles to report on more complex biological systems.

### Multimodal imaging

**Fig. 8.**
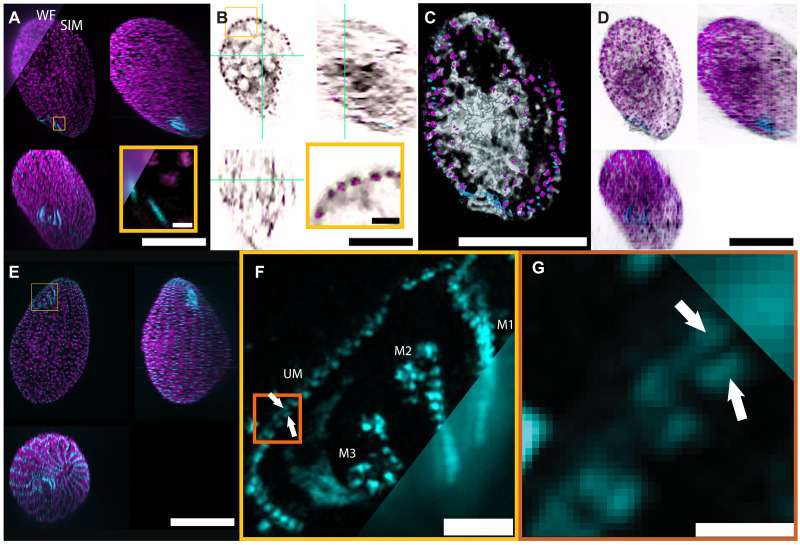
Multimodal imaging with SIM and FS-ODT. (**A**) Two-color SIM and pseudo–wide field (WF) MIPs of mitochondria (magenta) and basal bodies (cyan) in a fixed *Tetrahymena* cell. SIM achieves a better resolution of the mitochondria substructure, as well as the basal bodies near the OA. The coverslip is at the top of the maximum *y* projection (bottom left) and the left of the maximum *x* projection (top right). Scale bar, 20 μm. Inset (yellow) shows basal bodies and mitochondria near the OA in a single *z* plane. Scale bar, 1 μm. (**B**) Multimodal images in individual planes (blue-green lines). The RI exhibits similar morphology to the mitochondria. The inset (yellow) shows the colocalization of RI features with labeled mitochondria. Scale bar, 3 μm. (**C**) Multimodal image rendered through a 1.25-μm slice. Mitochondria cluster near the periphery of the cell. RI recapitulates much of the mitochondria distribution and additional structure inside the cell and near the OA. The RI is shown using an inverted color map relative to (B). Scale bar, 20 μm. (**D**) Combined SIM and RI (gray) MIPs after registration. The RI reveals the internal structure of the cell, including most likely vacuoles and the macronucleus. (**E**) SIM MIPs for a second *Tetrahymena* cell with OA better aligned to the optical axis. Scale bar, 20 μm. (**F**) MIP of the SIM basal body channel near the OA corresponding to the yellow ROI in (E). We identify the four membranelles: M1, M2, M3, and the UM. SIM resolves the double basal body structure of the UM, which is not visible in the wide field (bottom right). White arrows indicate a basal body pair. Scale bar, 2 μm. (**G**) Basal body pairs along the UM corresponding to the orange ROI in (F). Scale bar, 0.5 μm.

Last, we demonstrate the full multimodal imaging capability of our combined SIM and FS-ODT system by studying fixed *Tetrahymena* cells in solution (Fig. 8 and movie S7). We labeled mitochondria with Alexa Fluor 647 and basal bodies, which are 0.2 μm–by–0.5 μm cylindrical organelles that play a role in anchoring cilia to the cell body (*46*), with Alexa Fluor 488. We collected SIM *z*-stacks over 30 μm using 465- and 635-nm excitation lasers with the SIM pattern period chosen to allow a resolution enhancement factor of ~1.7. We reconstructed the super-resolution images using a Wiener filter approach implemented in our mcSIM package (*32*). The SIM reconstructions enhance the resolution of the mitochondria structures. We note that, as expected for many ciliates, the mitochondria are predominately localized around the cell periphery (*47*). The basal bodies are much sparser, and the SIM super-resolution enhancement is most noticeable near the OA, which is clearly visible in the MIPs (Fig. 8A). We include band reweighting using Gaussian suppression (*48*) in our SIM reconstruction to combine optical sectioning with super-resolution enhancement. The superior optical sectioning achievable in SIM is visible in Fig. 8A.

The *Tetrahymena* OA has been previously studied in considerable detail, particularly using electron microscopy (*46*). The OA includes four membranelles, often referred to as M1, M2, M3, and the undulating membrane (UM). The UM contains a double row of basal bodies arranged in a zipper-like pattern (*46*, *49*), which can be resolved in the SIM but not the wide field (Fig. 8, E and F). To examine the OA in Fig. 8 (E and F), we focus on a different cell than in Fig. 8 (A to D), choosing one with the OA better aligned to the optical axis.

We also image the *Tetrahymena* cells’ RI using FS-ODT with 347 illumination angles. We find that the RI reconstruction often shows similar structures to the labeled mitochondria (Fig. 8, B and C). In *Tetrahymena*, the mitochondria are often part of a larger network of organelles that exist at the cell cortex (*50*), and it is not clear whether it is the mitochondria or other members of this cortical network that provide the observed RI contrast. We observe additional internal structure, including low RI features that may be food vacuoles, and a larger, round, high-RI feature, which is most likely the macronucleus (Fig. 8D). In addition, we see high-RI structures near the OA that may be bunched oral cilia or fibril bundles (Fig. 8C, bottom left). RI features are more elongated along the axial direction than comparable SIM fluorescence features due to a combination of the lower NA of the FS-ODT imaging system and the optical sectioning in the SIM reconstruction algorithm. Some of the RI features visible in Fig. 8 (B and C) correspond to mitochondria visible in nearby *z* planes.

Because the SIM and ODT rely on separate views, with different spatial sampling, of the same sample, we register the two images by manually identifying ~20 morphological features that appear in both the RI and the mitochondria channels. Then, we estimate the 3D affine transformation that relates the images using the random sample consensus algorithm (RANSAC) (*51*) to reduce the effect of incorrectly identified control points. Using this transformation, we estimate the RI values on the SIM grid using linear interpolation and overlay the two images (Fig. 8C).

Previous efforts to combine diffraction tomography and SIM achieved single-color SIM operation (*20*, *27*, *52*, *53*). This study demonstrates combining multicolor SIM with ODT. Multimodal approaches combining fluorescence super-resolution with QPI can provide a wealth of information about morphology and dynamics together with molecular specificity (*54*). Combining SIM with knowledge of the RI distribution also opens up the possibility of joint inference, which could accurately correct for changes in the SIM pattern due to the RI distribution, potentially allowing super-resolution imaging deeper into samples (*55*, *56*).

## DISCUSSION

ODT is a powerful label-free 3D imaging approach with broad applications in biomedical imaging, biophysics, and soft matter physics. However, most efforts focus on improving ODT for applications specific to biomedical imaging. Biophysics and soft matter systems have very different imaging requirements, motivating our development of multiplexed FS-ODT pattern generation and reconstruction approaches.

We demonstrated that FS-ODT provides high-quality RI reconstructions of a variety of samples, including cells, 10-μm microspheres, diffusing colloids, and swimming bacteria. We present multiplexed illumination strategies to increase the number of illumination angles in a single image and expand the system’s field of view. Combined, both multiplexing strategies enable kilohertz-scale ODT over extended lateral fields of view.

Although the kilohertz-scale volumetric imaging is fast enough for many applications, FS-ODT imaging could be further accelerated by adopting alternative pattern generation hardware. For example, using faster DMD models that have pattern update rates up to 30 kHz could immediately accelerate the volumetric imaging rate by a factor of 3. Alternatively, acousto-optic deflectors or electro-optic modulators could accelerate pattern generation by one to two orders of magnitude, at the cost of complicating the off-axis holography reference generation. These fast speeds might be necessary for studying swimming in fast ciliates, which can move at speeds of up to 4000 μm s^−1^ (*57*) or studying the diffusion of single proteins (*58*).

Although FS-ODT already produces high-quality reconstructions in most circumstances, further improvements are possible by applying proximal gradient methods or deep learning approaches to unmix the off-axis holograms, denoise raw data, suppress coherent speckle, or perform phase unwrapping. Supervised learning approaches may improve the speed or quality of multiplexed reconstructions (*16*, *59*).

Alternatively, emerging deep-learning regularization approaches could be incorporated to improve FS-ODT reconstructions further. For example, DNN denoisers can be incorporated as priors in our existing FISTA framework using the plug-and-play prior or regularization by denoising approaches (*60*). Recent self-supervised approaches have achieved impressive results while preserving a physics-based forward model by using DNNs to parameterize the RI distribution, either as a pixelated image as in deep image prior approaches (*61*) or as a function of coordinates, as in neural field–based approaches (*62*). To account for motion blur, it may be possible to adapt neural field approaches to create a space-time model and infer dynamics during hologram acquisition (*63*).

We have also demonstrated multimodal imaging combining multicolor SIM with ODT, which could open exciting new approaches to event-driven smart microscopy (*64*). For example, FS-ODT could be used to continuously monitor a dynamic sample for behavior of interest, such as a microswimmer approaching a surface, and trigger SIM super-resolution imaging. This approach would limit photobleaching and phototoxicity, due to the near-infrared wavelength and low-imaging power used in FS-ODT, while retaining molecular information, which is vital for understanding biological systems.

We anticipate that FS-ODT will unlock challenging imaging regimes that have been difficult or impossible to explore. These include probing the ~100-Hz mechanical motion and hydrodynamics of cilia during swimming of protists such as *Tetrahymena* or exploring the behavior of microswimmers and active colloids in dense or complex 3D environments. It also extends the frontier of QPI to complex object tracking in dense environments and the exploration of highly dynamic systems.

## MATERIALS AND METHODS

### Computer control

The microscope is controlled using a custom computer package (*65*) and a GUI based on Napari-MicroManager, which relies on the MicroManager device drivers but replaces the Java GUI with Napari (https://github.com/QI2lab/napari-micromanager). The control computer is a Lenovo ThinkStation P620 running Microsoft Windows 10 Enterprise. This computer has a Ryzen Threadripper Pro 3945WX CPU (AMD) with 12 cores, 128-GB RAM, and a GeForce RTX 3090 GPU (NVidia) with 24 GB of memory. ODT patterns are preloaded on the DMD firmware, and the entire microscope acquisition is hardware triggered by a National Instruments DAQ (PCIe-6343). The Phantom camera includes 72 GB onboard memory and the 10-gigabit Ethernet option. It communicates with the PC using an X540-T2 10GbE card. A full 72 GB camera acquisition can be transferred to the computer in ~2 min.

### RI reconstruction

FS-ODT reconstructions were carried out using a custom Python code (*65*) running on Python 3.11.4 or 3.11.5 and CUDA toolkit 11.8.0. We implemented light scattering forward models and proximal gradient algorithms, harnessing CuPy for GPU acceleration and Dask for parallelization and orchestration. We rely on the RAPIDS cuCIM implementation of the total variation proximal operator, which is modeled on the scikit-image implementation, denoise_tv_chambolle. For weighted phase unwrapping during computing the Rytov field, we adapted the approach in (*66*) to run on the GPU.

Reconstructions were performed on either the experimental control computer described above or a custom-built computer running Ubuntu 20.04.6 LTS with an X99S SLI Plus motherboard (MSI), an i7-5820K CPU (Intel), 125-GB RAM, and an RTX A6000 GPU (NVidia) with 48 GB of memory.

### Preparation of *Tetrahymena* samples

For the samples in Fig. 1, *Tetrahymena* were cultured at room temperature in media containing 2% proteose peptone, 0.2% yeast extract, 0.012 mM FeCl_3_, 0.2% glucose, penicillin (100 U/ml), streptomycin (100 mg/ml), and amphotericin B (0.25 mg/ml). *Tetrahymena* were passaged to fresh media every 3 to 4 days. *Tetrahymena* were stained as described previously at room temperature unless noted otherwise (*67*). Briefly, ~2 × 10^6^ mid-log cells were washed with 10 mM Tris (pH 7.5), permeabilized in 0.25% TX-100 in PHEM for 30 s (60 mM Pipes, 25 mM Hepes, 10 mM EGTA, and 4 mM MgSO_4_), fixed in 1% paraformaldehyde in PHEM for 15 min, blocked in 3% bovine serum albumin (BSA) in PBS-T for 30 min [0.01% Tween 20, 130 mM NaCl, 2 mM KCl, 8 mM Na_2_HPO_4_, 2 mM KH_2_PO_4_, 10 mM EGTA, and 2 mM MgCl_2_ (pH 7.5)], stained with primary antibodies diluted in 3% BSA in PBS-T at 4°C overnight (glutamylated tubulin 1:1000, GT335, Adipogen, AG-20B-0020-C100 and centrin 1:1000, 20H5, MilliporeSigma, 04-1624), stained with secondary antibodies diluted in 3% BSA in PBS-T for 1 hour (1:1000 goat anti-mouse IgG2a Alexa Fluor 488, Invitrogen, A-21131 and 1:1000 goat anti-mouse IgG1 Rhoadmine Red X, Jackson ImmunoResearch, 115-005-205), and counterstained with 4′,6-diamidino-2-phenylindole (DAPI) (1 mg/ml) for 5 min. Samples were washed three times with phosphate-buffered saline (PBS) after fixation, primary antibody incubation, and secondary antibody incubation. Each wash lasted 5 min, and all centrifugations were carried out for 5 min at 250*g* in a swinging bucket centrifuge.

For the samples in Fig. 1B, we placed 100 μl of fixed *Tetrahymena* cells in PBS in a 40-mm-diameter cell culture dish (FluoroDish FD5040). For the samples in Fig. 1C, 10 μl of fixed *Tetrahymena* cells in PBS was placed on a round #1.5 coverslip of a diameter of 40 mm. A square #1 coverslip with 25-mm sides was dropped on top, and the chamber was sealed with epoxy (Devcon 2 Ton Epoxy, GLU-735.90). The electric field data were binned by a factor of 2 to reduce the memory required during reconstruction.

The samples used in Fig. 8 were prepped using a different procedure. One hundred microliters of *Tetrahymena thermophila* cells suspended in 10 ml of SuPP media was grown in 10-cm plates at room temperature for 3 days. After growing for 3 days, cell plates were poured into 15-ml conical tubes and centrifuged at 250*g* for 3 min to create a cell pellet. The supernatant was removed, and the cells were fixed in 10 ml of 100% methanol for 20 min at −20°C. After fixation, the tubes were moved to room temperature and the methanol was removed. The pellet had settled by gravity so centrifugation was not necessary. Cells were then permeabilized with a final concentration of 0.25% TX-100 in 1x PHEM and incubated in the permeabilization buffer for 10 min at room temperature. Once permeabilization was complete, the supernatant was removed and the cells were split into 1.7-ml Eppendorf tubes with 150 μl in each Eppendorf tube and 50 μl of sodium azide to prevent bacterial growth. Cells were blocked using 1 ml of PBS-T–BSA and incubated with the blocking buffer for 30 min. The PBS-T–BSA was then removed, and the primary antibodies were added. Both of the primary antibodies, ATP5A (ab14748) and 20H5 (04-1624), were diluted in PBS-T–BSA at a 1:500 ratio before being added to the cells. Cells were incubated with the primary antibodies overnight (~12 hours) at 4°C. The pellet settled via gravity overnight, and the supernatant was removed before adding 500 μl of PBS-T–BSA and incubating for 30 min to wash the cells. After washing, the PBS-T–BSA was removed and the secondary antibodies, GaR647 (A21244) and GaM 2a 488 (A21131), were prepared by diluting in PBS-T–BSA at a 1:500 ratio. The cells were left to incubate at room temperature for 2 hours. Once incubation in secondaries was complete, the secondary antibody solution was removed, and DAPI was added at a 1:50,000 ratio using 1x immunofluorescence (IF) PBS as a dilution buffer. Cells were incubated in the DAPI solution for 30 min and then washed twice with 500 μl of 1x IF PBS by adding the buffer and incubating for 20 min before removing. Cells were stored at 4°C.

To avoid photobleaching during SIM imaging, we diluted 2 μl of *Tetrahymena* solution in 40 μl of anti–bleaching imaging buffer composed of 2x SSC buffer (Invitrogen, AM9763), 50 mM tris-HCl (pH 8) (Thermo Fisher Scientific, AM9856), 10% (w/v) glucose (Sigma-Aldrich, DX0145-1), 2 μM Trolox (Sigma-Aldrich, 0.5 mg/ml), glucose oxidase (Sigma-Aldrich, G2133), and catalase (40 μg/ml; Sigma-Aldrich, C30) in 18-megohm water.

SIM reconstructions were carried out using mcSIM (*32*) with the Wiener parameter set to 0.6 and Gaussian suppression SD $0.4\times f_{\text{max}}$. For Fig. 8 (F and G), we additionally perform 30 iterations of combined Hessian and $\ell_{1}$ regularization with deconvolution and background subtraction turned off, fidelity set to 50 and sparsity set to 1.67 (*68*).

### Preparation of 10-μm PMSs

PMSs of a diameter of 10 μm (Thermo Fisher Scientific, FluoSpheres F8836) were sonicated for 20 min and then diluted by a factor of 10 in immersion oil (1.04699.0500, MilliporeSigma) with the RI in the range of 1.515 to 1.517. We spread 20 μl of the resulting emulsion on a #1.5 coverslip of a diameter of 40 mm with a pipette tip and left it uncovered overnight for the water to evaporate. Then, we dropped a #1 square 25 mm–by–25 mm coverslip on top and sealed the chamber with epoxy (Devcon 2 Ton Epoxy, GLU-735.90). After the epoxy dried, the sample was placed on the microscope, and a few drops of water were placed on the top coverslip to facilitate imaging with a water dipping objective.

### Preparation of diffusing microspheres in a water-glycerol mixture

PMSs of diameter 2R=1 μm (Thermo Fisher Scientific, FluoSpheres F8823) of a weight/volume of 0.02 g ml^−1^ were first sonicated for 20 min and then diluted by a factor of 10 in Milli-Q water. Ten microliters of this dilution was combined with 40 μl of Milli-Q water and 50 μl of glycerol. Ten microliters of this mixture was placed on a round #1.5 coverslip of a diameter of 40 mm. A square #1 coverslip with 25-mm sides was dropped on top, and the chamber was sealed with epoxy (Devcon 2 Ton Epoxy, GLU-735.90).

This experiment was performed on an earlier version of the apparatus. The detection objective was a 50× long-working distance air objective with NA = 0.55 (Mitutoyu, 378-805-3) and tube lens with a focal length of 200 mm (Thorlabs, ACT508-200-A-ML). The camera was a Prime BSI Express (Teledyne Photometrics) with 6.5-μm pixels and 1.8 RMS (root mean square) read noise. No beam expander was used after the detection tube lens. The effective pixel size was 0.130 μm. The frame rate was limited by the readout time of the camera.

The viscosity of this mixture is η = 0.0076 Pa s, and the density is $\rho_{\text{solvent}}=1.13\;g\;{\text{ml}}^{-1}$ at *T* = 22.5°C. The expected diffusion coefficient far from the wall is $D_{o}=k_{B}T/6\pi \eta R=0.0579\;{\mu m}^{2}\;s^{-1}$. The measured average diffusion coefficient is 0.0536 μm^2^/s. Deviations may come from differences in temperature and magnification from nominal values and the hydrodynamic interactions with the wall. The density of polystyrene is $\rho_{\text{bead}}=1.05\;g\;{\text{ml}}^{-1}$ and therefore the microspheres are buoyant in this mixture. We observe that the height distribution of the microspheres matches a Boltzmann distribution with the expected effective mass $m=\frac{4}{3}\pi R^{3}\left(\rho_{\text{bead}}-\rho_{\text{solvent}}\right)$.

After reconstructing the sample RI, we identified and localized the microspheres using a custom Python package, localize-psf (https://github.com/QI2lab/localize-psf). To identify candidate microspheres, we first applied a difference-of-Gaussian filter to suppress noise and background, then applied a maximum filter and selected pixels where the initial and maximum filtered images had the same values. We keep only points above a threshold. Next, we fit a region of interest (ROI) of size 4 μm by 2 μm by 2 μm around each candidate to a 3D Gaussian and only kept spots where the fit parameters fell within certain bounds. After localizing the microspheres at all time points, we tracked them using trackpy v0.6.1 (http://soft-matter.github.io/trackpy). To mitigate the effect of localization errors, we computed the MSDs using every eighth frame.

### Preparation of *E. coli* and microspheres

A culture of *E. coli* strain RP437, which is wild type for motility, was inoculated from a single colony and incubated overnight at 37°C in Lysogeny broth [tryptone (10 g liter^−1^), yeast extract (5 g liter^−1^), and NaCl (10 g liter^−1^)]. Cultures were diluted 1:100 in T-broth [tryptone (10 g liter^−1^) and NaCl (10 g liter^−1^)] and incubated at 33°C while shaking at 200 rpm, until reaching an OD_600_ (optical density at 600 nm) of 0.5. Cells were then centrifuged at 1200*g* for 7 min, washed, and resuspended in motility buffer [10 mM K_2_HPO_4_ and 0.1 mM EDTA (pH 7.5)]. The mobility buffer’s RI was 1.333 as measured using a refractometer (Krüss HR 901). The bacterial concentration was estimated to be 1.2 × 10^8^ cells/ml using a hemacytometer (Fisher Scientific, 0267151B).

PMSs of a diameter of 0.5 μm (Thermo Fisher Scientific, FluoSpheres F8813) of a weight/volume of 0.02 g ml^−1^ were first sonicated for 20 min and then diluted by a factor of 3 × 10^2^ with the motility buffer and *E. coli* mixture. Coverslips were cleaned with EtOH. A 120-μm tall chamber was prepared by placing a secure seal spacer (Electron Microscopy Science, catalog no. 70327-20S) on a round #1.5 coverslip of a diameter of 40 mm. Forty microliters of solution was placed in this chamber, and a square #1 coverslip with 25-mm sides was placed on top to close the chamber.

To quantify the bacteria swimming and identify the wobble angle, we trained a classifier to distinguish between *E. coli*, tracer particles, and background pixels using LabKit (*69*). Next, we labeled individual bacteria and identified their main axis using principal components analysis using scikit-image and tracked the bacteria with trackpy. To ensure that we tracked only swimming bacteria, we exclude any tracks shorter than 50 frames or that move a total distance of less than 4 μm. To generate the smoothed velocity, we first smoothed the centroid position by computing a rolling average over a window of size 15 and then computed the velocity using a second-order difference method (*70*).

### Preparation of diffusing microspheres in water

PMSs of a diameter of 1 μm (Thermo Fisher Scientific, FluoSpheres F8823) were sonicated for 20 min and then 3 μl was diluted with 97 μl of Milli-Q water. For the shorter chamber, 10 μl of the dilution was pipetted on a #1.5 coverslip of a diameter of 40 mm and a square #1 coverslip with 25-mm sides was dropped on top. The chamber was sealed with epoxy (Devcon 2 Ton Epoxy, GLU-735.90). The height of the fluid chamber was estimated to be ~10 μm from a fluorescence *z*-stack. For the taller chamber, we placed a 120-μm-thick spacer (Electron Microscopy Sciences 70327-20S) on the round coverslip, pipetted 40 μl of solution, and closed the chamber with the square coverslip. To achieve a DMD-limited frame rate, we cropped the camera chip to 960 pixels by 1040 pixels. We identified and tracked the PMS using the same procedure as for the PMS in the water-glycerol mixture.

## Supplementary Materials

### The PDF file includes:

Supplementary Text Figs. S1 to S6 Tables S1 and S2 Legends for movies S1 to S7 References
