# Supplementary material for: Fourier synthesis optical diffraction tomography for kilohertz rate volumetric imaging
Source: Sci Adv. 2025 Aug 13;11(33):eadr8004. doi: 10.1126/sciadv.adr8004 (PMC12346287; doi:10.1126/sciadv.adr8004)
Supplement: Supplementary file 1 — Supplementary Text Figs. S1 to S6 Tables S1 and S2 Legends for movies S1 to S7 References [file sciadv.adr8004_sm.pdf]

Science Advances
AAAS

Supplementary Materials for

Fourier synthesis optical diffraction tomography for kilohertz rate volumetric imaging

Peter T. Brown *et al.*

Corresponding author: Peter T. Brown, ptbrown1729@gmail.com;
Douglas P. Shepherd, douglas.shepherd@asu.edu

Sci. Adv. **11**, eadr8004 (2025)
DOI: 10.1126/sciadv.adr8004

The PDF file includes:

Supplementary Text
Figs. S1 to S6
Tables S1 and S2
Legends for movies S1 to S7
References

Other Supplementary Material for this manuscript includes the following:

Movies S1 to S7

Supplementary Text

S1 FS-ODT multiplexing validation with Mie theory

We validate our reconstruction approach by comparing with exact results using Mie theory (*77*). The Mie theory solution to scattering from a spherical dielectric particle expresses the field as a sum of vector spherical harmonics. Each spherical harmonic is a product of sinusoids, spherical Bessel functions, and associated Legendre polynomials. Although many Python packages calculate the expansion coefficients, few compute the electric fields. We rely on miepython (*78*) to compute the expansion coefficients and developed GPU accelerated Python code to calculate the Mie fields. Our code relies on CuPy functions where possible. Since the first order spherical Bessel functions j_n and their derivatives are not implemented in CuPy, we wrote a CUDA kernel which computes j_n efficiently using several different strategies depending on the value of x and the maximum index N. As usual, we exploit the three term recursion relationship relating spherical Bessel functions of different n values. To avoid the need to rerun this computation for $n = 1, ...N$, our routine provides the full sequence $j_1(x), ..., j_N(x)$. For $x \leq 0.1$ we calculate $j_n(x)$ according to the first four terms in its Taylor expansion. For $0.1 < x \leq N$, upward recursion is unstable for the $j_n(x)$, and so we use downward recursion and Miller's algorithm (*79*). For $x \geq N$ we apply upward recursion, since the j_n are oscillatory and this is stable (*80*). For an image size of 700×650 pixels, the CPU implementation runs in ~120 s while the GPU implementation runs in ~0.4 s.

Using Mie theory simulations, we validate that our multiplexed ODT patterns provide more information than non-multiplexed patterns and consequently produce superior reconstructions, compared with non-multiplexed patterns when the number of patterns is kept fixed (Figs. S1, S2, and S3). We compare results for 15 images using 10× multiplexing and without multiplexing, using two different non-multiplexed pattern sets (un-optimized and optimized). To generate simulated images, we first compute the Mie electric fields using the expansion discussed above for a sphere of diameter 10 µm and refractive index 1.59 in media with background refractive index 1.515, chosen to match a PMS microspheres in immersion oil. Our Mie routine outputs both the scattered and unscattered electric fields for a variety of initial plane wave incidence angles. To simulate multiplexing, we select a subset of fields, apply a phase shift to each, and then sum them. We

interfere the resulting electric field with a reference plane wave. Then we produce the detected intensity patterns by applying photon shot noise, camera gain (0.39 ADU/e$^-$) and offset (0 ADU), and camera readout noise (10 e$^-$). We keep the maximum photon number fixed at ~2000 for the final images. We reconstruct the simulated images following the same procedure as for the experimental data.

We find that the multiplexed images (Fig. S1) produce superior reconstructions to the non-multiplexed images (Figs. S2 and S3) for a variety of different regularization conditions. However, the degree of reconstruction enhancement depends on the object and the choice the non-multiplexed patterns. Note that unlike the comparison in Fig. 4, the number of images has been kept fixed rather than the number of plane waves (Fig. S1B, S2B, S3B).

We first consider non-multiplexed ODT where the pattern set is created by choosing one plane wave from each multiplexed pattern (Fig. S2). The resulting plane waves all lie in the $f_x > 0$ region of Fourier space. The RI reconstructions are elongated in the axial direction (Fig. S2A), showing a stronger missing cone effect than the multiplexed images (Fig. S1). The reconstructed RI is also skewed, with the elongation following a diagonal line, reflecting the compressed Fourier coverage provided by the pattern set (Fig. S1B). This effect is not present in the multiplexed patterns.

The 3D Fourier transforms of the recovered scattering potential also substantiate that multiplexing leads to an improved RI reconstruction. For all TV strengths, the $f_x - f_y$ Fourier space slice exhibits better infill for the multiplexed data in Fig. S1C compared with Fig. S2C. Similarly, the $f_x - f_z$ and $f_y - f_z$ slices of the multiplexed reconstruction scattering potential reflect the shape of the expected transfer function considerably better than the non-multiplexed reconstruction scattering potential.

We next consider non-multiplexed ODT where the pattern set is created by by choosing all of the plane waves from the first multiplexed pattern, and the first five from the fourth multiplexed pattern (Fig. S3). This pattern set provides better Fourier space coverage, and the resulting RI reconstructions are not as strongly affected by the missing cone as in Fig. S2. However, there are still important differences between these reconstructions and the multiplexed reconstructions of Fig. S1. In particular, the non-multiplexed data with $\tau_{\mathrm{tv}} = 10^{-3}$ is elongated along the axial direction compared with the multiplexed reconstruction, likely reflecting the weaker influence of the data fidelity term in the inverse problem for fewer plane waves. For weaker regularization strength,

the non-multiplexed RI reconstructions are skewed in the maximum-intensity projection along the x-direction (upper right), whereas the multiplexed reconstructions do not exhibit this effect. Additionally, the non-multiplexed reconstruction shows a more pronounced asymmetry for the two hemispheres above and below the central xy-plane compared with the multiplexed reconstruction.

To better understand the effect of TV regularization, we examine several different values $\tau_{\mathrm{tv}} = 10^{-3}, 10^{-4}, 0$. Other regularization terms are chosen similar to those we use for experimental data, specifically $n' \geq n_o$, $n'' = 0$, and $\tau_{\ell_1} = 10^{-4}$. For the multiplexed data, Fig. S1A, this relatively weak TV regularizer serves to smooth out some RI inhomogeneities that are retained from the structure of the electric fields. This smoothing effect starts to become noticeable at regularizer strength 10^{-3}. For the non-multiplexed data, the TV has a weaker effect since there are fewer density inhomogeneities to begin with.

S2 Optical diffraction tomography

Optical diffraction tomography was performed using a Mach-Zender interferometer, part of a bespoke multimodal microscope with quantitative phase imaging (FS-ODT) and multicolor fluorescence superresolution microscopy (structured illumination) capabilities (see Fig. S5 and table S2). Drawings and CAD files for custom parts are available online (*81*). The SIM portions of this microscope are described in previous work (*32*).

Up to 80 mW of 785 nm light with coherence length of ~50 m is generated using a volume-holographic-grating (VHG) stabilized laser (Thorlabs FPV785P). This light is divided using a polarizing beam splitter, and the two paths are coupled into separate 2 m long polarization-maintaining fibers (SMF1 and SMF2). One fiber is used to generate the ODT excitation light, and the other is used to generate the reference beam for the off-axis holography.

The reference beam is collimated with a molded aspheric lens of focal length 13.86 mm (C3, Thorlabs C560TME-B) and beam expanded with lenses of focal length 40 mm (L9, Thorlabs AC254-040-B-ML) and 300 mm (L8, Thorlabs AC508-300-AB-ML). It is combined with the reference beam on a d-mirror (Thorlabs BBD1-E03) near the focal plane of the 40 mm lens.

The excitation light is collimated with a molded aspheric lens of focal length 18.4 mm (C2, Thorlabs A280TM-B) and beam expanded by lenses with focal length 30 mm (LF, Thorlabs AC254-

030-AB-ML) and 125 mm (LG, Thorlabs LA1986-B-ML), resulting in a Gaussian beam with waist ~7 mm which is incident on the DMD.

The ODT patterns are generated from diffraction off a DMD (Texas Instruments DLP6500) with 7.56 µm pitch and 1920×1080 mirrors. We have discussed the details of our DMD geometry elsewhere (*32*). Let $\hat{\mathbf{x}}$ point along the long axes of the mirror grid and $\hat{\mathbf{y}}$ point along the short axis, both in the plane of the DMD face. Let $\hat{\mathbf{p}} = (\hat{\mathbf{x}} + \hat{\mathbf{y}})/\sqrt{2}$, $\hat{\mathbf{m}} = (\hat{\mathbf{x}} - \hat{\mathbf{y}})/\sqrt{2}$, and $\hat{\mathbf{z}}$ be the normal vector of the DMD surface pointing outwards. The DMD mirrors swivel about $\hat{\mathbf{p}}$ the axis and can be in two binary states, either $\gamma_{\pm} = \pm 12°$, which we will call the + and − states. The DMD chip is rotated so that the optical table normal points along $\hat{\mathbf{p}}$, ensuring that the principle diffraction occurs in the mz plane.

The initial DMD geometry was designed to enable 3 color SIM with excitation wavelength 465 nm, 532 nm, and 635 nm. For these three colors to all roughly meet the blaze condition, the DMD face normal $\hat{\mathbf{z}}$ makes an angle of $\theta_d \sim -21.2°$ with the optical axis. In the DMD coordinate system, the optical axis points along the $\sin\theta_d \hat{\mathbf{m}} + \cos\theta_d \hat{\mathbf{z}}$.

To achieve high-efficiency ODT, we align the 785 nm excitation light to approximately satisfy the blaze condition for the $(n_x, n_y) = (-3, 3)$ diffraction order when the mirrors are in the − state. The excitation light is incident at an angle of approximately $\theta_e = 2.42°$ so that $\hat{\mathbf{v}}_e = \sin\theta_e \hat{\mathbf{m}} - \cos\theta_e \hat{\mathbf{z}}$. The light diffracted into $(-3, 3)$ travels along $\theta_o = -23.48°$ so that $\hat{\mathbf{v}}_o = \sin\theta_o \hat{\mathbf{m}} + \cos\theta_o \hat{\mathbf{z}}$.

As discussed in the main text, we apply an additional "carrier frequency" to our patterns of frequency $\mathbf{f}_c = \frac{1}{4}\hat{\mathbf{x}} - \frac{1}{4}\hat{\mathbf{y}}$ 1/mirror. This produces additional diffraction about the $(-3, 3)$, and we have designed our system such that the $(-3, 3) + (1/4, -1/4)$ diffraction order travels along the optical axis and is nearly blazed. The perfectly blazed output direction for the − mirrors is $\sin\theta_b \hat{\mathbf{p}} + \cos\theta_b \hat{\mathbf{z}}$ for $\theta_b = -21.58°$. An aperture blocks all other diffraction orders. This specific carrier frequency is chosen to displace the beam as far as possible from orders of the form $(n/2, m/2)$, as we see substantial diffraction due to the + mirror states. Furthermore, this avoids diffraction along the $\hat{\mathbf{x}}$ and $\hat{\mathbf{y}}$ axes coming from a row of DMD mirrors beyond the active chip, which are fixed in the − state.

The DMD is in a conjugate plane to the back focal plane (Fourier plane) of the excitation and detection objectives. After light diffracts off of the DMD, it is relayed by a pair of imaging systems, the first using lenses of focal length 200 mm (L1, Nikon MXA20696) and 100 mm (L2,

Thorlabs AC508-100-A-ML), and the second using lenses of 400 mm (L3, Thorlabs AC508-400-A-ML) and 300 mm (L4, Thorlabs AC508-300-A-ML). After the Nikon tube lens, the NIR light is separated from the visible light with a dichroic mirror (SP1, Semrock FF750-SDi02-25x36). After the 100 mm achromat, they are recombined using a second identical dichroic mirror (SP3). An additional dichroic mirror (SP2) is arranged at a right angle to SP3 to prevent the differential $s-$ and $p-$phase shifts caused by a single mirror from affecting the polarization of the visible light (*82*). We align the polarization orthogonal to the table surface to avoid similar polarization degradation of the ODT beam by the epifluorescence dichroic for the fluorescence modality (DM2).

The ODT light is focused with an oil immersion objective (OBJ1, Olympus UPlanFL N 100x NA 1.3), interacts with the sample, and is collected using a water objective (OBJ2, Olympus LUMPLFLN60XW) and a 180 mm tube lens (L6, Thorlabs AC508-180-AB-ML). The image is then magnified by a factor of 3 using a relay composed of a 100 mm (L7, Thorlabs AC508-100-B-ML) and a 300 mm (L8, Thorlabs AC508-300-AB-ML) lens. The light is imaged onto a Phantom camera (Cam2, VEO-1010L-72G-M) which has 1280×960 pixels, pixel size 18 µm, quantum efficiency ~51 % at 785 nm, read-noise 10.5 e^- RMS and gain of 0.39 ADU/e^-, measured using the approach of (*83*). The effective pixel size is 0.1 µm. The maximum frame rate for the full chip is 8420 frame/s, but it can be increased by cropping the chip.

DMD efficiency

The expected peak DMD diffraction efficiency into any order (n_x, n_y) is ~50 %, limited by the reflectivity of the DMD mirrors, the fraction of the chip the mirrors cover, the transmissivity of the DMD window, and diffraction physics. Using the carrier frequency, we expect that efficiency into the ±1 carrier orders are each ~25 % of the power in the 0th order. We expect the power in 0th order is ~18 % of the power that would be diffracted if the mirrors were all in the $-$ state. Taken together, we estimate the DMD diffraction efficiency into the desired order is ~2 %.

The fiber coupling efficiency is ~50 %. Additionally, since the fluorescence modality of our microscope operates at visible wavelengths, most of the optical coatings are optimized for visible light. Thus, only ~75 % of light diffracted from the DMD reaches the objectives. The two objective lenses have a combined transmissivity of ~50 %. The efficiency from the DMD to the camera is

thus ~30 %,

As only a small fraction of the DMD mirrors are used to generate a plane wave, this further reduces the efficiency. We adjust the magnification between the back focal plane and the DMD so that the pupil radius is approximately the same size as the DMD along its narrow dimension. As the magnification factor is $M = 0.625$, the detection objective pupil radius is $R_p = \text{NA}\, f/0.625 = 4.8\,\text{mm}$ at the DMD. For a spot pattern of radius R and assuming the laser power is distributed uniformly over the pupil, we expect the number of photons per second that strike the camera is

$$\frac{80\,\text{mW}}{hc/\lambda} \times \frac{\pi R^2}{\pi R_p^2} \times (0.5 \times 0.02 \times 0.3) \approx 2 \times 10^{11}\,\text{photon/s}. \tag{S1}$$

for $R = 10 \times 7.56\,\mu\text{m}$. Here, we divide the terms into incident power, geometric efficiency, and transmission/diffraction efficiency.

For patterns of radius 10 mirrors, our simulations show that the beam waist in the imaging plane is ~10 µm (Fig. S4E), and putting this all together, for an imaging time of 100 µs we expect to collect about

$$N = 2 \times 10^{11}\,\text{photon/s} \times \frac{\left(20\,\mu\text{m} \times \frac{1}{180}\right)^2}{\pi\,(10\,\mu\text{m})^2} \times 100\,\mu\text{s} \times \text{QE} \approx 400\,\text{photon} \tag{S2}$$

per pixel, where the quantum efficiency of the camera is QE ~ 50 % at 785 nm.

During typical operation, the power of a single ODT beam generated by a spot pattern with 10 mirror diameter is 1.5 µW after diffracting from the DMD, and 0.2 µW after passing through the objective. The reference beam power is 1.05 µW.

Pattern fidelity

Previous ODT approaches using binary DMD patterns have resulted in lower-quality reconstruction than gray-scale approaches due to the unwanted additional diffraction orders introduced by the DMD's square binary pixels. Our previous structured illumination microscopy work addressed similar issues (*32*). In both cases, these spurious diffraction orders arise when using large-scale periodic patterns covering the face of the DMD. In this case, our small spot patterns lead to much smaller contributions from these orders, which are mitigated by Fourier broadening (Fig. S4B).

DMD non-planarity

Unlike in many DMD imaging applications, we do not place the DMD orthogonal to the optical axis (Fig. S4A). This compromise improves the diffraction efficiency by satisfying the blaze condition. In our geometry, the effect of this tilt is minor.

The tilt introduces a shift in the focus of the plane waves across the DMD face. At the pupil radius, the DMD z-shift is at most $R_p \sin\theta_D \sim 1.73$ mm. As the z-magnification is $M_z = M^2 = 0.39$ the shift in the objective back-focal plane is 0.67 mm. The shift must be compared with the depth-of-focus of the beam in the back focal plane, which we estimate using the Rayleigh range. For a plane wave with $w_o = 10\,\mu$m in the focal plane, the waist in the objective BFP is ~37 µm corresponding to a Rayleigh range of $\pi w_o^2/\lambda \sim 5.5$ mm, which is one order of magnitude larger than the focal shift.

The tilt also introduces deformations in the transformation between the position on the DMD face and the frequency of the beam. For example, there is some shear, and a ring pattern on the DMD maps to an oval in frequency space. The precise effects can be calculated using the approach of (*32*) described in section 3 of the supplemental methods.

System stability

Due to the relatively long beam path (> 2 m) used in our multimodal microscope setup, we observe several sources of instability in our system and ODT patterns, which we correct for computationally during ODT reconstruction. Specifically, we correct for (1) phase drift between the reference arm and the imaging arm, (2) frequency instability of the ODT patterns, and (3) position instability of the ODT patterns.

To correct for (1), we determine the complex factor relating the image electric fields to a single background electric field using a least-squares fit. To correct for (2), we determine the location of the Fourier peaks versus time by fitting the Fourier transform of the hologram image to a Gaussian in the vicinity of each peak. To correct for (3), we register images using the Fourier transform of the absolute value of the electric field. Empirically, we find that using the absolute value of the electric field is superior to using either the Fourier transform of the intensity or the Fourier transform of the electric field. The improvement is likely due to the rejection of background fluctuations that do not

involve the interference pattern and taking the absolute value removes the need to consider phase fluctuations of the electric field.

The stability of our system over ~4 s is shown in Fig. S6. Over this short time span the relative phase between the imaging and reference beam drifts by $\sim 2\pi$ radians, the spatial position drifts by <50 nm, and the beam frequency drifts by $<4 \times 10^{-3}$ µm^{-1}. Note that the Fourier space pixel sizes are $df_x = 7.81 \times 10^{-3}$ µm^{-1} and $df_y = 10.42 \times 10^{-3}$ µm^{-1}, so the frequency drift is sub-pixel.

S3 Iterative reconstruction with proximal gradient methods

As FS-ODT multiplexing introduces a more challenging computational image reconstruction problem requiring a more complex approach, we briefly discuss some commonly used reconstruction approaches. In 1969, Wolf introduced optical diffraction tomography using a Born approximation formulation (*8*). In this formulation, the forward model describing the electric field after interacting with a given RI distribution is linear. This linearity makes solving the inverse problem, i.e. inferring the RI based on the observed electric fields, particularly simple. Devaney realized that the Rytov approximation is better suited for biological sample (*9*), and various technical improvements have expanded the range of validity and quality of reconstructions (*84–91*). However, imaging thicker and higher-contrast samples inevitably introduces multiple scattering, which the linear Born and Rytov approximations do not include. To address multiple scattering, a variety of multi-slice reconstruction approaches have been developed that rely on the beam-propagation model (BPM) (*92–96*). However, the BPM entails the paraxial approximation, motivating the development of more accurate forward models, including the split-step non-paraxial (SSNP) model (*38*), more sophisticated Born approximation approaches (*97–99*), and HyPM (*100*). Most approaches consider forward scattering only, which is implicit in the layer-by-layer multi-slice forward models. Other approaches account for backscattering using the Lippman-Schwinger equation (*101–103*), but these are generally more computationally expensive than multi-slice models. Machine learning approaches are increasingly employed to accelerate and denoise RI reconstruction (*59*). In this work, we primarily rely on the BPM or the SSNP combined with an initial guess generated using a demultiplexed low-resolution Rytov approximation approach to infer RI information from FS-ODT data.

We treat refractive index reconstruction as a regularized minimization problem and solve it using

the fast iterative shrinkage-thresholding algorithm (FISTA) (*37*). As usual in FISTA, we attempt to minimize a function which is the sum of two terms, a loss function and a regularization function $\mathcal{L}(x) + g(x)$, where $\mathcal{L}$ is differentiable and has a Lipschitz-continuous gradient with Lipschitz constant L, and g is convex. We iteratively update the proposed solution, x_t, starting from $t = 0$, $q_0 = 1$, the step-size γ, and a starting guess x_o

$$\begin{aligned} y_t &= \text{prox}_{\gamma_t} \left[x_{t-1} - \gamma \nabla \mathcal{L}(x_{t-1})\right] \\ q_t &= \frac{1 + \sqrt{1 + 4q_{t-1}^2}}{2} \\ x_{t+1} &= y_t + \frac{q_{t-1} - 1}{q_t} \left(y_t - y_{t-1}\right). \end{aligned}$$

In the last step the convergence is accelerated by including a momentum term and the structure of q_t is chosen to change the convergence from $O(1/t)$ to $O(1/t^2)$.

The proximal operator for g,

$$\text{prox}_\gamma(z) = \text{argmin}_x \left\{ g(x) + \frac{1}{2\gamma} |x - z|^2 \right\}, \tag{S3}$$

determines a new object which is near to the initial value but better satisfies the regularization. When an explicit form of or fast algorithm for computing the proximal operator is known, this process is efficient, as in the case of total variation (*104*), ℓ_1, or ℓ_2 norms. In this work we choose a regularization function which enforces smoothness and sparsity

$$g(x) = \tau_{o,\text{tv}} TV(x) + \tau_{o,\ell_1} \|x\|_1, \tag{S4}$$

where TV is the 3D isotropic TV operator. Since the TV proximal problem is typically solved using an iterative procedure (*104*), fully evaluating eq. S3 is expensive. Instead, we typically limit the number of iterations to 5. Additionally, we optionally impose RI constraints $n' \geq n_o$ and $n'' \geq 0$ through projection. In our implementation, the user sets the proximal parameter $\tau = \tau_o \gamma$, which is simpler to work with and more intuitive than τ_o.

In some cases, we adopt the plug-and-play (PnP) prior approach (*105*) and replace eq. S3 by applying a denoiser directly. In this work, we only explore simple examples such as applying a median filter to the RI.

The total loss, $\mathcal{L}(x_t) + g(x_t)$, is non-increasing when the step-size $\gamma \in [0, 2/L]$. If the Lipschitz constant is not known, a line-search strategy can determine the step-size at each iteration by

reducing an initial step-size until the Lipschitz condition is locally satisfied (algorithm 1). This requires additional calculation of $\mathcal{L}(y)$ and $\mathcal{L}(x)$.

Data: Proposed object x

Data: Initial step-size γ_o

Data: Step-size adjuster $\alpha < 1$

$\gamma \leftarrow \gamma_o$;

$y \leftarrow \text{prox}\left[x - \gamma \nabla \mathcal{L}(x)\right]$;

while $\mathcal{L}(y) > \mathcal{L}(x) + \nabla \mathcal{L}(x) \cdot (y - x) + \frac{1}{2\gamma} \|y - x\|_2^2$ **do**

$\quad \gamma \leftarrow \alpha\gamma$;

$\quad y \leftarrow \text{prox}\left[x - \gamma \nabla \mathcal{L}(x)\right]$;

end

Algorithm 1: Line-search algorithm for setting the step-size

In ODT, we measure a sequence of electric fields $\mathbf{\Psi}^{(i,d)}$, where $i = 1, ..., M$ indexes the incident angles, derived from off-axis holography and define our loss function by

$$\mathcal{L} = f \mathcal{L}_E + (1 - f) \mathcal{L}_I \tag{S5}$$

$$\mathcal{L}_E(n) = \frac{1}{2M} \sum_{i=1}^{M} \left\| \mathbf{\Psi}^{(i)} - \mathbf{\Psi}^{(i,d)} \right\|_2^2 \tag{S6}$$

$$\mathcal{L}_I(n) = \frac{1}{2M} \sum_{i=1}^{M} \sum_{j=1}^{N} \left\| \left| \Psi_j^{(i)} \right| - \left| \Psi_j^{(i,d)} \right| \right\|^2 \tag{S7}$$

where $f \in [0, 1]$ describes the relative strength of the phase-sensitive ($\mathcal{L}_E$) and phase-insensitive ($\mathcal{L}_I$) components of the loss function, $\mathbf{\Psi}^{(i)}(n)$ is the forward model describing the predicted electric field as a function of n, $\|\cdot\|_2$ is the ℓ_2 norm, and N is the number of pixels in a single electric field. Note that many different loss functions can be defined for the phase-insensitive portion, and in fact the most natural one might seem to involve $|\Psi|^2$, however previous work has demonstrated better convergence using eq. S7 (*106*).

To perform FISTA, we require the gradient of the loss function with respect to the refractive index. The loss function, $\mathcal{L} : \mathbb{R}^N \times \mathbb{R}^N \to \mathbb{R}$, can be interpreted either using the real and imaginary parts of the refractive index as components of a vector, or as complex numbers. Since $\mathcal{L}$ is not holomorphic, if we regard the domain as $\mathbb{C}^N$ we must compute the gradient using Wirtinger

derivatives (*35*), defined by $\partial_z = \frac{1}{2}(\partial_{z'} - i\partial_{z''})$ and $\partial_{z^*} = \frac{1}{2}(\partial_{z'} + i\partial_{z''})$, where z' and z'' are the real and imaginary parts of z respectively. In this formalism, the gradient used in FISTA is $\nabla = 2\partial_{z^*}$. Computing this derivative, which only acts directly on the forward model, we find (suppressing the beam angle index)

$$\nabla_{n_b(z_l)}\mathcal{L} = \sum_a \frac{\partial \mathcal{L}}{\partial \Psi_a^*}\left[\frac{\partial \Psi_a}{\partial n_b(z_l)}\right]^* \tag{S8}$$

$$\partial_{\mathbf{\Psi}^*}\mathcal{L}_E = \left[\mathbf{\Psi} - \mathbf{\Psi}^{(d)}\right] \tag{S9}$$

$$\partial_{\mathbf{\Psi}^*}\mathcal{L}_I = \left[|\mathbf{\Psi}| - \left|\mathbf{\Psi}^{(d)}\right|\right] \odot \frac{\mathbf{\Psi}}{|\mathbf{\Psi}|} \tag{S10}$$

where b and l index the xy- and z-position of the voxels respectively and we have used the chain rule $\partial_{n^*}(\mathcal{L} \circ \Psi) = \partial_{\Psi^*}\mathcal{L}\,(\partial_n \Psi)^* + \partial_\Psi \mathcal{L}\,(\partial_{n^*}\Psi)$ and the fact $\partial_{n^*}\Psi = 0$.

Due to the FS-ODT pattern generation strategy, our illumination beams typically do not cover the entire field of view and exhibit decreasing intensity near the edges. In regions with less intensity, the influence of the loss function decreases and the effect of the regularization increases. Therefore, to achieve a higher quality reconstruction over a larger field of view it is helpful to normalize the loss function by the electric field. We optionally replace the loss function with

$$\mathcal{L}_E = \frac{1}{2M}\sum_{i=1}^{M}\sum_{j=1}^{N}\frac{\left|\Psi_j^{(i)} - \Psi_j^{(i,d)}\right|^2}{\left|\Psi_j^{(i,d)}\right|^2 + \alpha^2} \tag{S11}$$

$$\mathcal{L}_I = \frac{1}{2M}\sum_{i=1}^{M}\sum_{j=1}^{N}\frac{\left|\left|\Psi_j^{(i)}\right| - \left|\Psi_j^{(i,d)}\right|\right|^2}{\left|\Psi_j^{(i,d)}\right|^2 + \alpha^2}, \tag{S12}$$

where α is a regularization parameter that prevents division by small numbers where the electric field is noise dominated. The denominator does not depend on the forward model, so the loss function gradients need only be divided elementwise by the denominator to account for this change.

Linear scattering models

As usual we suppose light interacts with a spatially varying refractive index according to the scalar Helmholtz equation and work with phasors carrying $\exp(i\omega t)$ time dependence,

$$\left[\nabla^2 + k^2 n^2(\mathbf{r})\right] E(\mathbf{r}) = 0. \tag{S13}$$

where $k = 2\pi/\lambda n_o$.

Additionally we define the scattering potential V

$$V(\mathbf{r}) = -\left(\frac{2\pi}{\lambda}\right)^2 \left[n^2(\mathbf{r}) - n_o^2\right] \tag{S14}$$

We suppose that our sample is illuminated by a sequence of plane waves and the ith plane wave has frequency $\mathbf{f}^{(i)}$

$$E^{(i,o)} = \exp\left[-i2\pi\mathbf{f}^{(i)} \cdot \mathbf{r}\right] \tag{S15}$$

where $2\pi\left|\mathbf{f}^{(i)}\right| = k$.

In the Born approximation, valid when the cumulative phase shift of the beam is $\lesssim \pi/4$ (*107*), the 2D Fourier transform of the scattered electric field gives the scattering potential along a spherical cap in 3D Fourier space

$$\tilde{E}^{(i,s)}(f_x, f_y) = \frac{1}{2i \times 2\pi f_z(f_x, f_y)} \tilde{V}(f_x - f_x^{(i)}, f_y - f_y^{(i)}, f_z - f_z^{(i)}) \text{ (Born)} \tag{S16}$$

The Rytov approximation is an alternate approach which is usually more accurate for biological samples. In this approximation, the scattering potential is related to the Rytov phase $\psi(\mathbf{r})$,

$$E^{(i,s)}(\mathbf{r}) = E_o(\mathbf{r})\left(e^{\psi^{(i)}(\mathbf{r})} - 1\right) \tag{S17}$$

$$\psi^{(i)}(\mathbf{r}) = \log\left|\frac{E^{(i)}(\mathbf{r})}{E^{(i,bg)}(\mathbf{r})}\right| + i \text{ unwrap}\left\{\text{angle}\left[E^{(i)}(\mathbf{r})\right] - \text{angle}\left[E^{(i,bg)}(\mathbf{r})\right]\right\} \tag{S18}$$

$$\tilde{\psi}^{(i)}(f_x - f_x^{(i)}, f_y - f_y^{(i)}) = \frac{1}{2i \times 2\pi f_z(f_x, f_y)} \tilde{V}(f_x - f_x^{(i)}, f_y - f_y^{(i)}, f_z - f_z^{(i)}) \text{ (Rytov)} \tag{S19}$$

The Rytov approximation is valid when $n^2(\mathbf{r}) - n_o^2 \gg |\nabla\psi(\mathbf{r})|^2 k^2$ (*9*).

In this case, it is more convenient to work with the scattering potential than the refractive index, and the loss function (eq. S6) and its gradient are

$$\mathcal{L}(V) = \frac{1}{N}\frac{1}{2M}\sum_{i=1}^{M}\sum_{j=1}^{N}\left|\left(F^{(i)}\tilde{V}\right)_j - \tilde{\mathbf{\Psi}}_j^{(i)}\right|^2$$

$$\nabla_{\tilde{V}}\mathcal{L} = \frac{1}{N}\frac{1}{M}\sum_i \left(F^{(i)}\right)^\dagger \left(F^{(i)}\tilde{V} - \tilde{\mathbf{\Psi}}^{(i)}\right)$$

where $\tilde{\mathbf{\Psi}}^{(i)}$ is the scattered field or the Rytov phase depending on the approximation, $F^{(i)}$ is the forward model linear operator for the ith angle which connects the 3D Fourier transform of the

scattering potential, $\tilde{V}$, to $\tilde{\mathbf{\Psi}}^{(i)}$. The second factor of $1/N$ converts this to the loss in real-space accounting for the Fourier transform. Since the forward model is linear, a Lipschitz constant for the loss function is proportional to the largest eigenvalue of $F^t F$ which can be computed using a singular value decomposition or the power iteration algorithm.

Multi-slice models

Suppose that we can rewrite the Helmholtz equation (eq. S13) by reparameterizing the electric field as Ψ and rewriting the differential operator as the sum of two terms, where A describes the effect of the background refractive index n_o and B captures the effect of spatially varying refractive index perturbations,

$$\partial_z \Psi = (A + B)\, \Psi. \tag{S20}$$

Formally the solution is a path-ordered exponential, but for small enough δz,

$$\Psi(\delta z) \approx \exp\left[(A + B)\delta z\right] \Psi(0) \tag{S21}$$

$$\approx \exp\left[A\delta z\right] \exp\left[B\delta z\right] \Psi(0). \tag{S22}$$

For convenience we define $P = \exp\left[A\delta z\right]$ and $Q = \exp\left[B\delta z\right]$. Corrections to eq. S22 are given by the Baker-Campbell-Hausdorff formula, and the first correction is proportional to the commutator $[A, B]$. By construction, this vanishes when $n = n_o$, and we expect it and higher order commutators to be small when the refractive index perturbation is small.

We can propagate an initial field through a volume by discretizing it into layers of thickness δz and iteratively applying eq. S22

$$\mathbf{\Psi} = F(PQ)^{(k-1)} \ldots (PQ)^{(0)} \mathbf{\Psi}^{(0)} \tag{S23}$$

Here F describes model operations beyond the final refractive index plane, $\Psi^{(m)}$ is the intermediate field before the mth voxel, and Ψ is the detected field.

For models considered here, Q is local in the sense that the only dependence on $n(z_l)$ is in $Q^{(l)}$, and thus

$$\frac{\partial \Psi_a}{\partial n_b(z_l)} = \left(F(PQ)^{(k-1)} \ldots (PQ)^{(l+1)} P^{(l)} \frac{\partial Q^{(l)}}{\partial n_b(z_l)} \Psi^{(l)} \right)_a \tag{S24}$$

$$W^{(l+1)} = F(PQ)^{(k-1)} \ldots (PQ)^{(l+1)}, \tag{S25}$$

where the index a in the first equation indicates the ath component of the vector. Typically the structure of ∂Q allow us to simplify this expression. For example, in the BPM Q is diagonal due to the fact it does not mix the field at different $\mathbf{r}$ positions. For the SSNP, Q is local but mixes the derivative and field at the same position.

Beam-propagation model (BPM)

In the BPM, the Helmholtz equation is put in the form of eq. S20 by first making the paraxial approximation (*94*). Here we take $\Psi = E$ and the model is defined by

$$P\Psi = \mathcal{F}_\perp^{-1}\left\{\exp\left[i\delta z\sqrt{k^2 - k_x^2 - k_y^2}\right] \times \mathcal{F}_\perp\{\Psi\}(k_x, k_y)\right\} \tag{S26}$$

$$Q^{(l)}\Psi = \exp\left[ik_o\delta z\frac{n(x,y,z_l) - n_o}{\eta}\right] \times \Psi \tag{S27}$$

$$F\Psi = \mathcal{F}_\perp^{-1}\left\{H(k_x,k_y) \times \exp\left[i\delta z_f\sqrt{k^2 - k_x^2 - k_y^2}\right] \times \mathcal{F}_\perp\{\Psi\}(k_x, k_y)\right\} \tag{S28}$$

where H is the coherent transfer function, δz_f is the final distance the beam propagates, and $k_o = 2\pi/\lambda$. η is the obliquity factor which is taken to be 1 in most cases, but taking $\eta = 1/\cos\theta$ improves accuracy (*35*).

Following eqs. S8 and S24 the derivatives are

$$\frac{\partial Q^{(l)}_{cd}}{\partial n_b(z_l)} = \frac{ik_o\delta z}{\eta}\exp\left[ik_o\delta z\frac{n_c(z_l) - n_o}{\eta}\right] \times \delta_{cd} \times \delta_{bd} \tag{S29}$$

$$\frac{\partial \Psi_a}{\partial n_b(z_l)} = \frac{ik_o\delta z}{\eta}\left[F(PQ)^{(k-1)}...(PQ)^{(l+1)}\right]_{ab}\Psi_b^{(l+1)} \tag{S30}$$

$$\nabla_{n(z_l)}\mathcal{L} = -\frac{ik_o\delta z}{\eta}\left[\left(F(PQ)^{(k-1)}...(PQ)^{(l+1)}\right)^\dagger \partial_{\Psi^*}\mathcal{L}\right] \odot \left(\Psi^{(l+1)}\right)^* \tag{S31}$$

$$\nabla_n\mathcal{L} = \left[\left(\mathcal{F}_\perp^{-1}\right)^\dagger\{\partial_{\mathbf{\Psi}^*}\mathcal{L}\}\right] \odot \left[\exp\left(i\delta z_f\sqrt{k^2 - k_x^2 - k_y^2}\right) \times \mathcal{F}_\perp\left\{\mathbf{\Psi}^{(k)}\right\}\right]^* \tag{S32}$$

Split-step non-paraxial model (SSNP)

This model is extensively discussed elsewhere (*35,38*) and we briefly discuss it here for convenience. To rewrite the Helmholtz equation in the form of eq. S20 we must work with a vector of the electric field and its first derivative. The physical intuition behind this is as follows. Suppose we know the electric field in a single plane and wish to know its value everywhere. We can decompose the field

into Fourier modes, but we cannot distinguish forward and backwards traveling plane waves at the same lateral spatial frequency. We need additional information to untangle these two contributions: e.g. the magnetic field or the axial derivative of the electric field $\partial_z E$. Therefore the propagation operator must act on both the field and its derivative.

In this case the forward model is

$$\Psi = \begin{pmatrix} E \\ \partial_z E \end{pmatrix} \tag{S33}$$

$$P\Psi = \mathcal{F}_\perp^{-1}\left\{\begin{pmatrix} \cos k_z\delta z & \frac{1}{k_z}\sin k_z\delta z \\ -k_z \sin k_z\delta_z & \cos k_z\delta_z \end{pmatrix}\mathcal{F}_\perp\{\Psi\}\right\} \tag{S34}$$

$$Q^{(l)}\Psi = \begin{pmatrix} 1 & 0 \\ k_o^2\left[n_o^2 - n^2(x,y,z_l)\right]\delta z & 1 \end{pmatrix}\Psi \tag{S35}$$

$$F\Psi = \mathcal{F}_\perp^{-1}\left\{\begin{pmatrix} \frac{1}{2} & \frac{1}{2}\frac{1}{ik_z} \end{pmatrix} H(k_x,k_y) \times P(\delta z_f)\Psi\right\}. \tag{S36}$$

When we write Q as a matrix following eq. S24 we combine the structure of the $\mathbf{r}_\perp$ index and the field/derivative index. Let i and j index the position $\mathbf{r}_\perp$ and field/derivative respectively and define the composite index $c = 2i + j$. Following eqs. S8 and S24, the derivatives are

$$\frac{\partial Q_{cd}}{\partial n_b} = -2k_o^2\,\delta z\, n \times \delta_{c,2b+1}\,\delta_{d,2b} \tag{S37}$$

$$\frac{\partial E_a}{\partial n_b(z_l)} = -2k_o^2\,\delta z\, \times \left[F(PQ)^{(k-1)}...(PQ)^{(l+1)}P^{(l)}\right]_{a,2b+1} n_b\,\Psi_{2b}^{(l)} \tag{S38}$$

$$\nabla_n\mathcal{L} = -2k_o^2\,\delta z\left[\left(DF(PQ)^{(k-1)}...(PQ)^{(l+1)}P^{(l)}\right)^\dagger \partial_{\Psi^*}\mathcal{L}\right] \odot n^*(z_l) \odot \left(\mathbf{E}^{(l)}\right)^*. \tag{S39}$$

where in the last line $\Psi_{2b} = E_b$ and D is a projection operator which only retains the derivative index.

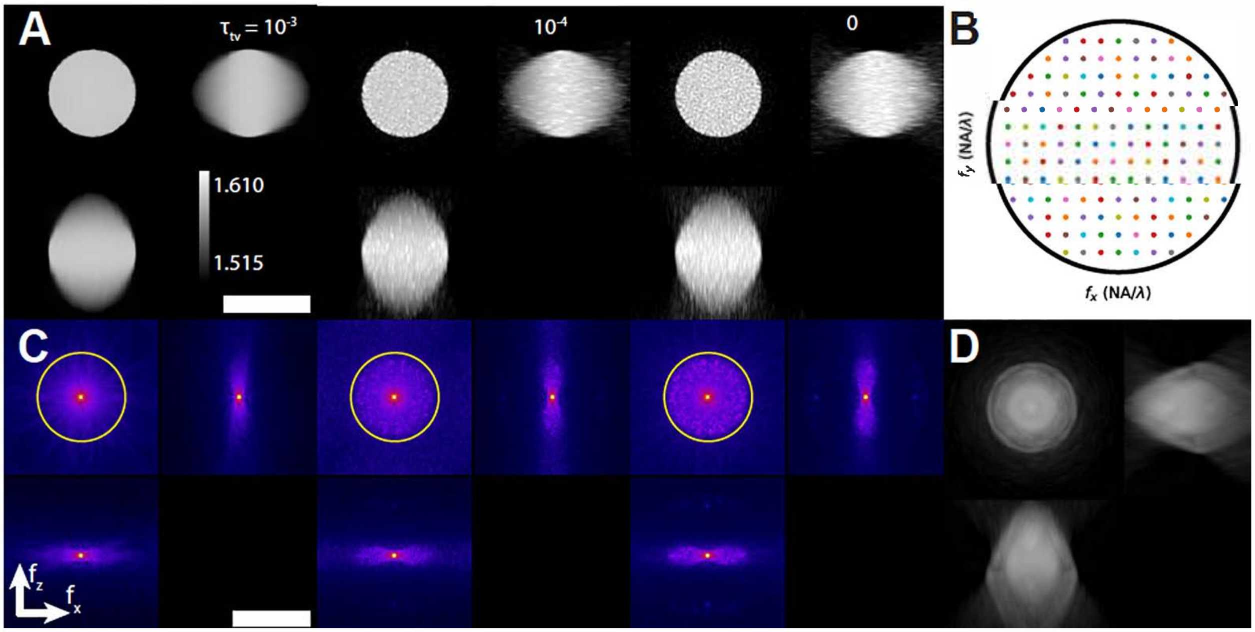

Figure S1: **Refractive index reconstruction using Mie theory and multiplexed ODT**. **A**. Maximum intensity projections of refractive index reconstruction of a simulated 10 µm diameter sphere using 10× multiplexing for TV regularization strengths of 10^{-3}, 10^{-4}, and 0 (left to right). The axial refractive index reconstruction is slightly distorted due to missing cone artifacts and the limited excitation and detection numerical aperture of 1. Scale bar 10 µm. **B**. Plane wave frequencies used in the multiplexed patterns. Frequencies used in a given pattern are assigned the same color. Black circle represents the pass-band of the system. **C**. Magnitude of the 3D Fourier transforms of the scattering potentials corresponding to the RI reconstructions shown in a. The orthogonal images shows slices along the $f_z = 0$ (upper left), $f_y = 0$ (lower left), and $f_x = 0$ (upper right) planes. Magnitudes are displayed using a gamma of 0.25. The maximum recoverable spatial frequency information in the weak-scattering approximations is illustrated in yellow. Scale bar 5 μm^{-1} **D**. Real part of the initial refractive index guess generated using the low-resolution Rytov demultiplexing approach.

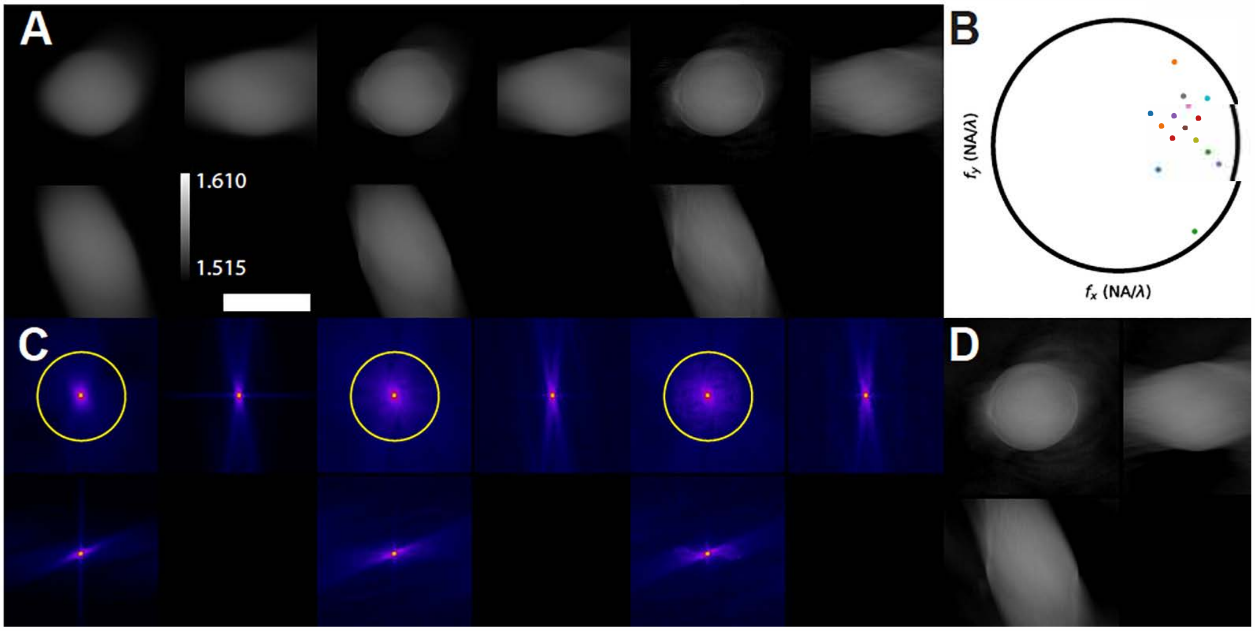

Figure S2: Refractive index reconstruction using Mie theory and non-multiplexed ODT, pattern set I: un-optimized. **A**. Maximum intensity projections of refractive index reconstruction of a simulated 10 µm diameter sphere without multiplexing. The limited angular information produces an inferior reconstruction compared with Fig. S1. Regularizaton parameters correspond with columns in Fig. S1. Scale bar 10 µm. **B**. Plane wave frequencies used in the non-multiplexed patterns. Black circle represents the pass-band of the system. **C**. Magnitude of the 3D scattering potentials corresponding to the RI reconstructions shown in a. **D**. Real part of the initial refractive index guess generated using the Rytov approximation.

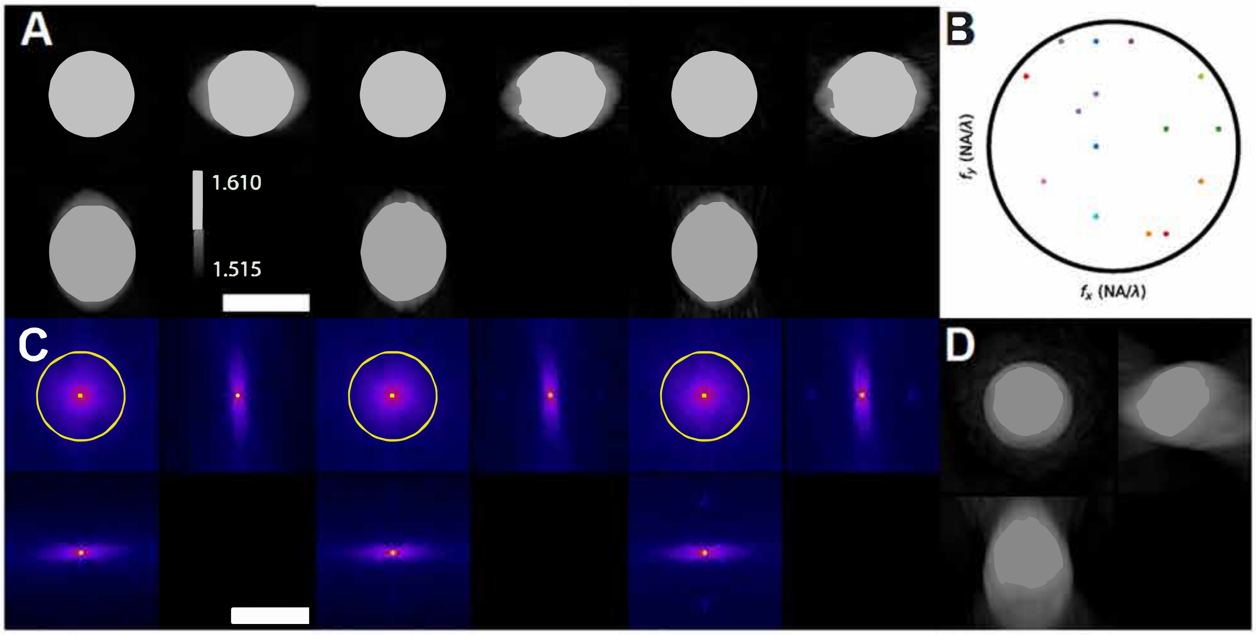

Figure S3: **Refractive index reconstruction using Mie theory and non-multiplexed ODT, pattern set II: optimized**. **A**. Maximum intensity projections of refractive index reconstruction of a simulated 10 µm diameter sphere without multiplexing. Regularizaton parameters correspond with columns in Fig. S1. Scale bar 10 µm. **B**. Plane wave frequencies used in the non-multiplexed patterns. Black circle represents the pass-band of the system. **C**. Magnitude of the 3D scattering potentials corresponding to the RI reconstructions shown in a. **D**. Real part of the initial refractive index guess generated using the Rytov approximation.

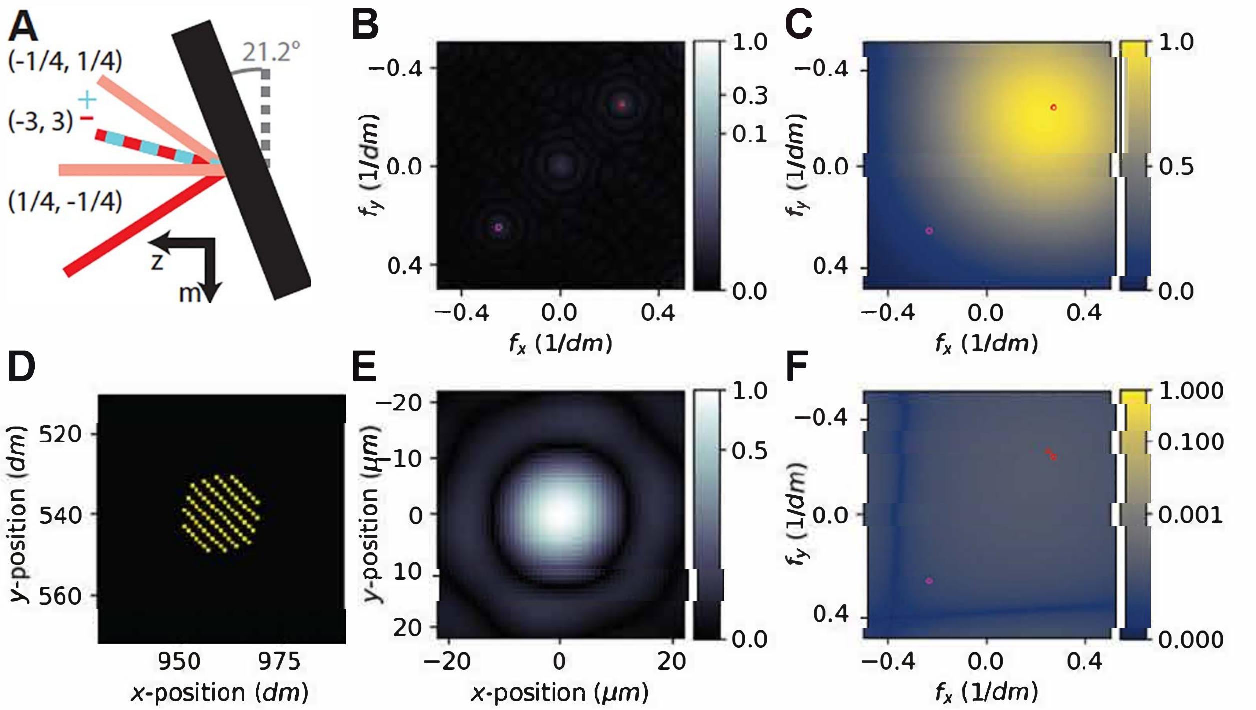

Figure S4: DMD geometry and pattern simulation. **A**. DMD geometry showing the incident NIR light (red) and the main diffraction orders for the + (cyan) and – (red) mirrors and the positive and negative diffraction at the carrier frequencies (pale red) **B**. Magnitude squared of the diffracted electric field versus frequency, $|E(f)|^2$. The diffracted weight is concentrated around the positive (red) and negative (magenta) carrier frequency diffraction orders **C**. Blaze envelope for – mirrors. **D**. Sample DMD pattern with $R = 10$ mirror show mirrors in the – state (yellow) and the + state (black) **E**. Pattern in sample plane with measured waist $w_o = 9.3\,\mu$m **F**. Blaze envelope for the + mirrors. The strength of this diffraction is suppressed by $\sim 10^{-5}$ compared with the – mirrors.

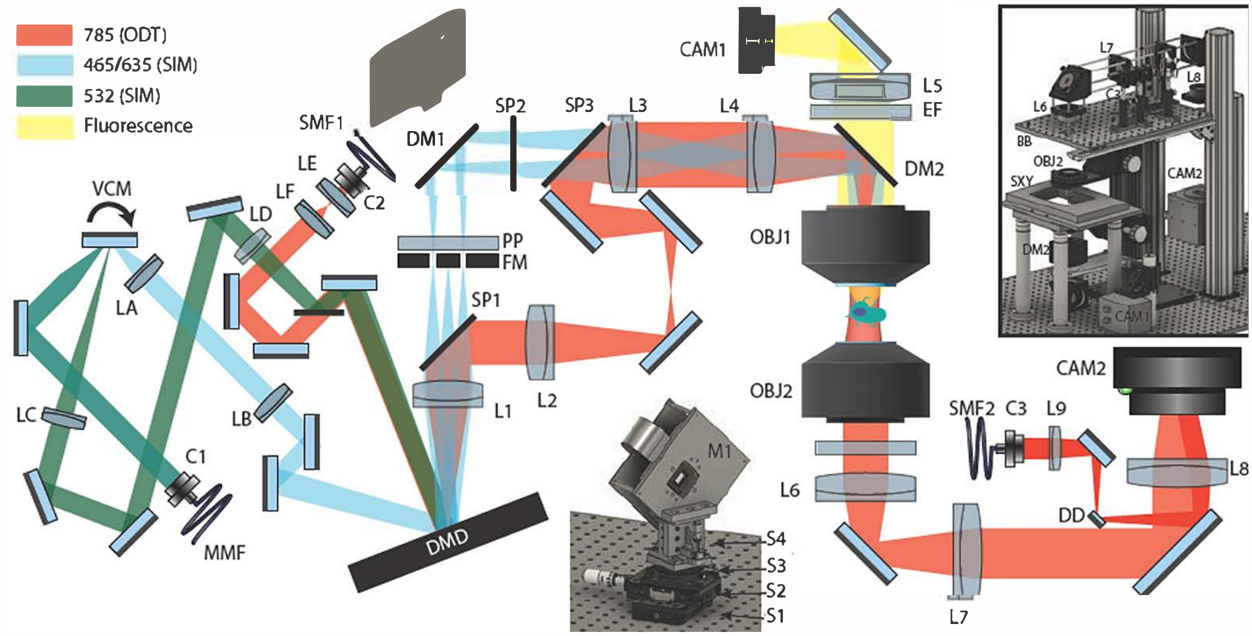

Figure S5: **Combined SIM and FS-ODT beam path**. SIM excitation light is emitted by a multimode fiber (MMF) and coupled toward the DMD. Different input directions for the three wavelengths are selected by adjusting the voice-coil mirror (VCM). The SIM excitation beams are relayed from the DMD to the sample using two relays formed by L1 and L3, and L4 and OBJ1. Beam filtering and polarization is accomplished in the Fourier plane after L1. Fluorescence light is collected on CAM1. ODT imaging light is emitted from a polarization maintaining single mode fiber (SMF1) and directed to the DMD. ODT light is split from the SIM by a short pass filter (SP1), passes through an extra lens (L2) and is recombined on SP3. ODT light passes through the sample, is collected by OBJ2, and is beam expanded onto CAM2. The reference beam light is emitted from SMF2 and coupled into the ODT beam path using a d-mirror. Insets show custom parts include a dichroic mirror holder machined to connect to a kinetic mirror mount (H1, upper left), the DMD mount (lower center), and the microscope body and breadboard supporting the reference beam (upper right). The ODT beam path is described in detail in section S2 and the components used are described in table S2.

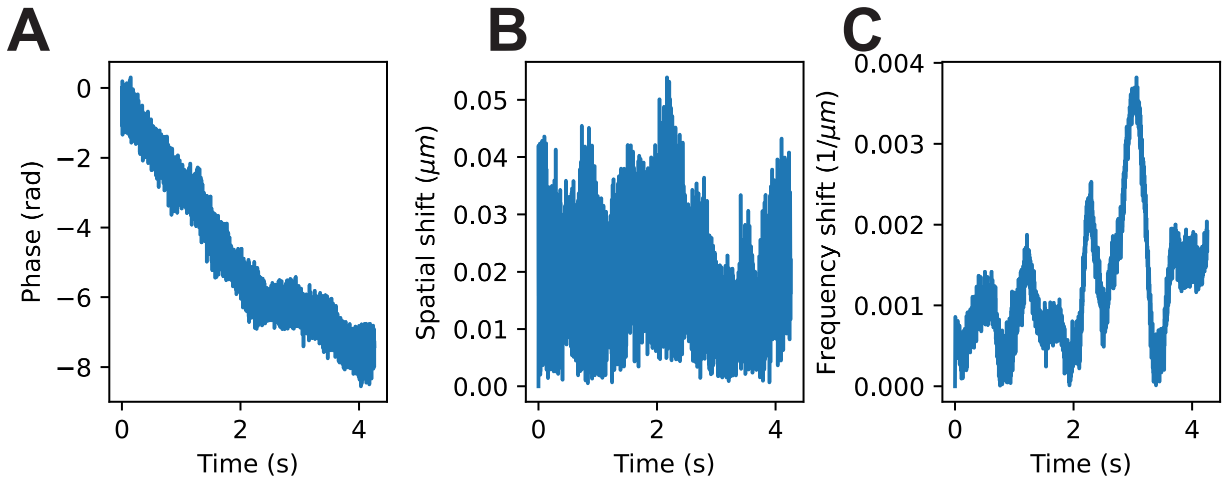

Figure S6: **System stability**. **A**. Exemplary phase stability plot from the first pattern from the 10 µm bead data. Phases are unwrapped **B**. Exemplary pattern position stability for the same pattern as A. **C**. Exemplary pattern frequency stability for one frequency in the pattern in A.

Table S1: **ODT reconstruction parameters**. Here we provide details about the ODT patterns and reconstruction parameters used in all data sets presented above. The pattern parameters are M, the number of images, N_p, the number of plane wave patterns, and N_t, the number of time lapse points collected. Rate is the volumetric image acquisition rate and exp is the exposure time for each off-axis hologram. The reconstruction parameters are the fraction of electric field loss function, f, the regularization parameters τ_{tv} and τ_{ℓ_1}, the allowed values for the imaginary part of the RI, n'', and the voxel size of the reconstruction grid, $dz \times dxy$.

sample	$M/N_p/N_t$	rate	exp	model	n_o	f	regularization	batch	$dz \times dxy$ / FOV ($z \times y \times x$)
Tetrahymena<br>Fig. 1B	331 / 331 / 1	–	1 ms	Rytov	1.333	1	$\tau_{\text{tv}} \le 0.1$<br>$\tau_{\ell_1} \le 0.01$<br>$n'' \ge 0$	1	0.869×0.202 µm / $32.2 \times 47.6 \times 60.9$ µm
Tetrahymena<br>Fig. 1C	145 / 145 / 1	–	600 µs	BPM	1.333	1	$\tau_{\text{tv}} \le 3 \times 10^{-10}$<br>$\tau_{\ell_1} = 0$<br>$n'' \ge 0$	3	0.87×0.2 µm / $26.97 \times 50.4 \times 56$ µm
10 µm PMS 1×<br>Fig. 4	147 / 147 / 1	–	400 µs	SSNP	1.515	1	$\tau_{\text{tv}} = 7.8 \times 10^{-4}$<br>$\tau_{\ell_1} = 2.3 \times 10^{-5}$<br>$n' \ge n_o$<br>$n'' = 0$	4	0.1×0.1 µm / $25.1 \times 70 \times 70$ µm
10 µm PMS 3×<br>Fig. 4	49 / 147 / 1	–	300 µs	SSNP	1.515	1	$\tau_{\text{tv}} = 1.6 \times 10^{-3}$<br>$\tau_{\ell_1} = 4.7 \times 10^{-5}$<br>$n' \ge n_o$<br>$n'' = 0$	4	0.1×0.1 µm / $25.1 \times 70 \times 70$ µm
10 µm PMS 10×<br>Fig. 4	15 / 150 / 1	–	150 µs	SSNP	1.515	0.1	$\tau_{\text{tv}} \le 0.1$<br>$\tau_{\ell_1} = 0$<br>$n' \ge n_o$<br>$n'' = 0$	2	0.1×0.1 µm / $25.1 \times 70 \times 70$ µm
10 µm PMS 19×<br>Fig. 4	8 / 152 / 1	–	75 µs	SSNP	1.515	0.01	$\tau_{\text{tv}} \le 0.1$<br>$\tau_{\ell_1} = 0$<br>$n' \ge n_o$<br>$n'' = 0$	2	0.1×0.1 µm / $25.1 \times 70 \times 70$ µm
1 µm PMS<br>Fig. 5B<br>Video 1	11 / 11 / 10 000	6.05 Hz	3 ms	Rytov	1.407	1	$\tau_{\text{tv}} = 0$<br>$\tau_{\ell_1} = 0$<br>$n'' \ge 0$	11	1.75×0.376 µm / ~$54 \times 67 \times 79$ µm
1 µm PMS<br>Fig. 5D<br>Video 2	11 / 11 / 2000	6.05 Hz	3 ms	Rytov	1.407	1	$\tau_{\text{tv}} = 0$<br>$\tau_{\ell_1} = 0$<br>$n'' \ge 0$	11	1.75×0.376 µm / ~$54 \times 67 \times 79$ µm
0.5 µm PMS<br>& *E. coli*<br>Fig. 6C,D<br>Video 3<br>Video 4	11 / 11 / 1000	143 Hz	600 µs	Rytov	1.334	1	$\tau_{\text{tv}} \le 3 \times 10^{-2}$<br>$\tau_{\ell_1} \le 1 \times 10^{-2}$<br>$n'' \ge 0$	11	0.435×0.196 µm / $25.6 \times 25.3 \times 26.5$ µm

sample	$M/N_p/N_t$	rate	exp	model	n_o	f	regularization	batch	$dz \times dxy$ / FOV ($z \times y \times x$)
1 µm PMS<br>Fig. 7A-F<br>Video 5	8 / 608 / 1000	1.032 kHz	75 µs	BPM	1.333	1	$\tau_{\text{tv}} \leq 10^{-2}$<br>$\tau_{\ell_1} \leq 3 \times 10^{-4}$<br>$n'' = 0$	3	0.5×0.1 µm / $40.5 \times 96 \times 104$ µm
1 µm PMS<br>Fig. 7G<br>Video 6	8 / 608 / 1000	1.032 kHz	75 µs	BPM	1.333	1	$\tau_{\text{tv}} \leq 10^{-2}$<br>$\tau_{\ell_1} \leq 10^{-4}$<br>$n'' = 0$	3	0.5×0.1 µm / $70.5 \times 96 \times 104$ µm
Tetrahymena<br>Fig. 8<br>Video 7	347 / 347 / 1	–	1.5 ms	BPM	1.333	1	$n' \geq n_o$<br>$n'' = 0$<br>$(1, 3, 3)$ median	20	1×0.1 µm / $47 \times 96 \times 104$ µm
10 µm Mie 10×<br>Fig. S1	15 / 150 / 1	–	–	SSNP	1.515	1	$\tau_{\text{tv}} = 10^{-3}, 10^{-4}, 0$<br>$\tau_{\ell_1} = 10^{-4}$<br>$n' \geq n_o$<br>$n'' = 0$	3	0.1×0.1 µm / $25.1 \times 70 \times 65$ µm
10 µm Mie 1×<br>Fig. S2	15 / 15 / 1	–	–	SSNP	1.515	1	$\tau_{\text{tv}} = 10^{-3}, 10^{-4}, 0$<br>$\tau_{\ell_1} = 10^{-4}$<br>$n' \geq n_o$<br>$n'' = 0$	3	0.1×0.1 µm / $25.1 \times 70 \times 65$ µm
10 µm Mie 1×<br>Fig. S3	15 / 15 / 1	–	–	SSNP	1.515	1	$\tau_{\text{tv}} = 10^{-3}, 10^{-4}, 0$<br>$\tau_{\ell_1} = 10^{-4}$<br>$n' \geq n_o$<br>$n'' = 0$	3	0.1×0.1 µm / $25.1 \times 70 \times 65$ µm

Table S2: Parts list corresponding to Fig. S5 and section S2. Parts which are not separated by lines are part of a single unit, *e.g.* an optic and an optics mount.

Label	Part	Manufacturer	Comments
	OEM 470nm-2W	Lasever	Blue SIM source. Measured wavelength ~465 nm
	LSR532NL-500	Lasever	Green SIM source
	LSR635-500	Lasever	Red SIM source. Diode laser, 500 mW
	FPV785P<br>LM14S2<br>LDC205C<br>TED200C	Thorlabs<br>Thorlabs<br>Thorlabs<br>Thorlabs	NIR laser, 100 mW, coherence length 50 m<br>14-pin butterfly laser diode mount<br>current controller<br>temperature controller
	WPMH05M-780	Thorlabs	half-wave plate for dividing ODT reference and imaging arms
	PBS122	Thorlabs	polarizing beamsplitter cube
	C060TMD-B	Thorlabs	mounted aspheric collimator $f = 9.6$ mm<br>Couple light into to SMF1 and SMF2
		custom	fiber shaker for despeckling MMF (*108*)
MMF	M103L05	Thorlabs	150×150 µm square core, NA 0.39
	AC254-30-A-ML	Thorlabs	collimated output from MMF
C1	AC254-30-A-ML	Thorlabs	
VCM	MR-15-30	Optotune	controlled through Python API
LA	CLS-SL	Thorlabs	scan lens
LB	AC508-400-A-ML	Thorlabs	
LC	AC508-075-AB-ML	Thorlabs	green beam expander
LD	AC508-500-A-ML	Thorlabs	green beam expander
SMF1<br>C2<br>LE<br>LF	P3-780PM-FC-2<br>A280TM-B<br>AC254-030-AB-ML<br>LA1986-B-ML	Thorlabs<br>Thorlabs<br>Thorlabs<br>Thorlabs	polarization maintaining patch cable, 2 m long, MFD 5.3 µm<br>mounted aspheric collimator, $f = 18.4$ mm<br>ODT beam expander<br>ODT beam expander
DMD<br>M1<br>S1<br>S2<br>S3<br>S4	DLP6500FYE<br><br>XR25C<br>XR25C<br>PR01<br>MVS010	Texas Instruments<br>custom<br>Thorlabs<br>Thorlabs<br>Thorlabs<br>Thorlab	DLP Light Crafter 6500, 1920×1080 mirrors with 7.56 µm pitch<br>DMD mount. Drawing available (*81*)<br>DMD x-stage<br>DMD y-stage<br>DMD rotation stage<br>DMD z-stage
L1	MXA20696	Nikon	collect diffracted light from DMD
SP1	FF750-SDi02-25x36	Semrock	separate SIM and ODT<br>750 nm edge short-pass dichroic beamsplitter

Label	Part	Manufacturer	Comments
FM		custom	Fourier mask, 3D printed using Form2 printer
			6 $\sim$1 mm diameter holes
	CXYZ-1	Thorlabs	FM xy-adjustment
PP	colorPol vis 500 BC4 CW01	Codixx	six element azimuthal ("pizza") polarizer
	CXY2	Thorlabs	PP xy-adjustment
DM1	zt473/532/633rdc-uf3	Chroma	Matched mirror for DM2 mounted on H1 and kinetic mirror mount
H1	WFA2002	Thorlabs	Four custom cutouts are machined so that flat spacers of equal height may be inserted and H1 can be attached to a kinematic mirror mount. Drawing available (*81*)
SP2	FF750-SDi02-25x36	Semrock	matched for SP3
L2	AC508-100-A-ML	Thorlabs	
SP3	FF750-SDi02-25x36	Semrock	recombine SIM and ODT light
L3	AC508-400-A-ML	Thorlabs	
L4	AC508-300-A-ML	Thorlabs	
	CFB1500	Thorlabs	microscope stand
DM2	zt473/532/633rdc-uf3	Chroma	
	WFA4111	Thorlabs	dovetail adapter
	WFA2002	Thorlabs	DM2 holder
	CSA1002	Thorlabs	DM2 support
Em	LF405-488-532-635-B-OMF	Semrock	emission filter. The full filter set is describe here
Em	ZET457NF	Chroma	
L5	SWLTU-C	Olympus	fluorescence arm tube lens
CAM1	Orca Flash4.0 v2	Hamamatsu	1.96 ADU/e^- gain, 2 e^- RMS readout noise
	C11440-22CU		0.71 QE at 590 nm, 6.5 µm pixel
OBJ1	UPLFLN100x	Olymus	100x, NA 1.3, FN 26.5 oil immersion UPlanFL
	ZFM1020	Thorlabs	OBJ1 support and height adjustment
	Nano F-200S	Mad City Labs	piezo z-stage
SXY	MicroDrive MCL-μD1803	Mad City Labs	xy-stage
OBJ2	LUMPLFLN60XW	Olympus	60x, NA 1.0, FN 26.5, WD 2mm, water dipper objective
	CXY2	Thorlabs	OBJ2 xy-adjustment
	ZFM1020	Thorlabs	OBJ2 support and height adjustment
BB	CSA3000	Thorlabs	breadboard for ODT reference arm combination
			Custom machined holes to connect with threaded rods
L6	AC508-180-AB-ML	Thorlabs	ODT path tube lens
L7	AC508-100-B-ML	Thorlabs	ODT relay lens # 1
L8	AC508-300-AB-ML	Thorlabs	ODT relay lens # 2

Label	Part	Manufacturer	Comments
CAM2	VEO-1010L-72G-M	Phantom	0.39 ADU/e^- gain, 10 e^- RMS read noise<br>0.62 QE at 650 nm, 18 µm pixel
SMF2	P3-780PM-FC-2	Thorlabs	see SMF1
C3	C560TME-B	Thorlabs	mounted aspheric collimator $f = 13.86$ mm, NA 0.18
L9	AC254-040-B-ML	Thorlabs	this and L8 form a beam expander for the ODT reference arm
DD	BBD1-E03	Thorlabs	d-mirror for combining reference beam

Caption for Movie S1. Hindered diffusion of 1 µm **polystyrene microspheres in water-glycerol mixture.** Maximum intensity projections of the refractive index of 1 µm polystyrene microspheres undergoing hindered diffusion in a water-glycerol mixture. Scale bar 20 µm.

Caption for Movie S2. Hindered diffusion of densely packed 1 µm **polystyrene microspheres in water-glycerol mixture** Maximum intensity projections of the refractive index of 1 µm polystyrene microspheres undergoing hindered diffusion in a water-glycerol mixture. Scale bar 20 µm.

Caption for Movie S3. Maximum intensity projections of 0.5 µm **polystyrene microspheres and swimming *E. coli*.** Orthographic XY, XZ, and YZ projections (left). Projection of the body axis (blue) and average velocity (red) are shown.

Caption for Movie S4. Orthographic projection of 0.5 µm **polystyrene microspheres and swimming *E. coli*.** Animated version of Figure 6A.

Caption for Movie S5. Freely diffusing 1 µm **polystyrene microspheres in water.** 1 µm polystyrene microspherein a 10 µm tall chamber filled with water imaged at a 1.032 kHz volumetric frame rate using multiplexed FS-ODT. Scale bar 10 µm.

Caption for Movie S6. Freely diffusing densely packed 1 µm **polystyrene microspheres in water.** Maximum intensity projections of the refractive index of 1 µm polystyrene microspheres diffusing in a 120 µm tall chamber filled with water imaged at a 1.032 kHz volumetric frame rate using multiplexed FS-ODT. Scale bar 20 µm.

Caption for Movie S7. Multimodal FS-ODT and SIM image of a Tetrahymena cell. Multimodal image of a Tetrahymena cell showing the RI (white) and SIM fluorescence (cyan and magenta). Cyan shows basal bodies and magenta mitochondria. First, we display a fly-through the volume, starting from the coverslip and progressing up through the sample rendering 1.25 µm thick sections. Next, we fly backwards through the sample, displaying a projection of all RI and fluorescence data down to the current slice. Finally, we rock the sample back and forth, illustrating the 3D data set from different viewing angles.

REFERENCES AND NOTES

1. P. P. Laissue, R. A. Alghamdi, P. Tomancak, E. G. Reynaud, H. Shroff, Assessing phototoxicity in live fluorescence imaging. *Nat. Methods* **14**, 657–661 (2017).

2. B. Javidi, A. Carnicer, A. Anand, G. Barbastathis, W. Chen, P. Ferraro, J. W. Goodman, R. Horisaki, K. Khare, M. Kujawinska, R. A. Leitgeb, P. Marquet, T. Nomura, A. Ozcan, Y. K. Park, G. Pedrini, P. Picart, J. Rosen, G. Saavedra, N. T. Shaked, A. Stern, E. Tajahuerce, L. Tian, G. Wetzstein, M. Yamaguchi, Roadmap on digital holography [Invited]. *Opt. Express* **29**, 35078–35118 (2021).

3. Y. Park, C. Depeursinge, G. Popescu, Quantitative phase imaging in biomedicine. *Nat. Photonics* **12**, 578–589 (2018).

4. W. Xu, M. H. Jericho, I. A. Meinertzhagen, H. J. Kreuzer, Digital in-line holography for biological applications. *Proc. Natl. Acad. Sci. U.S.A.* **98**, 11301–11305 (2001).

5. P. Girshovitz, N. T. Shaked, Fast phase processing in off-axis holography using multiplexing with complex encoding and live-cell fluctuation map calculation in real-time. *Opt. Express* **23**, 8773–8787 (2015).

6. K. Lindfors, T. Kalkbrenner, P. Stoller, V. Sandoghdar, Detection and spectroscopy of gold nanoparticles using supercontinuum white light confocal microscopy. *Phys. Rev. Lett.* **93**, 037401 (2004).

7. K. Mallery, J. Hong, Regularized inverse holographic volume reconstruction for 3D particle tracking. *Opt. Express* **27**, 18069–18084 (2019).

8. E. Wolf, Three-dimensional structure determination of semi-transparent objects from holographic data. *Optics Commun.* **1**, 153–156 (1969).

9. A. J. Devaney, Inverse-scattering theory within the Rytov approximation. *Opt. Lett.* **6**, 374–376 (1981).

10. V. Lauer, New approach to optical diffraction tomography yielding a vector equation of diffraction tomography and a novel tomographic microscope. *J. Microsc.* **205**, 165–176 (2002).

11. F. Charrière, A. Marian, F. Montfort, J. Kuehn, T. Colomb, E. Cuche, P. Marquet, C. Depeursinge, Cell refractive index tomography by digital holographic microscopy. *Opt. Lett.* **31**, 178–180 (2006).

12. W. Choi, C. Fang-Yen, K. Badizadegan, S. Oh, N. Lue, R. R. Dasari, M. S. Feld, Tomographic phase microscopy. *Nat. Methods* **4**, 717–719 (2007).

13. S. A. Alexandrov, T. R. Hillman, T. Gutzler, D. D. Sampson, Synthetic aperture fourier holographic optical microscopy. *Phys. Rev. Lett.* **97**, 168102 (2006).

14. G. Zheng, R. Horstmeyer, C. Yang, Wide-field, high-resolution Fourier ptychographic microscopy. *Nat. Photonics* **7**, 739–745 (2013).

15. K. Lee, K. Kim, J. Jung, J. H. Heo, S. Cho, S. Lee, G. Chang, Y. J. Jo, H. Park, Y. K. Park, Quantitative phase imaging techniques for the study of cell pathophysiology: From principles to applications. *Sensors* **13**, 4170–4191 (2013).

16. B. Ge, Y. He, M. Deng, M. H. Rahman, Y. Wang, Z. Wu, Y. Yang, C. Kuang, C. H. N. Wong, M. K. Chan, Y.-P. Ho, L. Duan, Z. Yaqoob, P. T. C. So, G. Barbastathis, R. Zhou, xSCYTE: Express single-frame cytometer through tomographic phase. arXiv:2202.03627 [physics. optics] (2022).

17. M. Lee, H. Jeong, C. Lee, M. J. Lee, B. R. Delmo, W. D. Heo, J. H. Shin, Y. K. Park, High-resolution assessment of multidimensional cellular mechanics using label-free refractive-index traction force microscopy. *Commun. Biol.* **7**, 115 (2024).

18. H. Hugonnet, Y. W. Kim, M. Lee, S. Shin, R. H. Hruban, S. M. Hong, Y. K. Park, Multiscale label-free volumetric holographic histopathology of thick-tissue slides with subcellular resolution. *Adv. Photonics* **3**, 026004 (2021).

19. J. Li, A. C. Matlock, Y. Li, Q. Chen, C. Zuo, L. Tian, High-speed in vitro intensity diffraction tomography. *Adv. Photonics* **1**, 066004 (2019).

20. D. Dong, X. Huang, L. Li, H. Mao, Y. Mo, G. Zhang, Z. Zhang, J. Shen, W. Liu, Z. Wu, G. Liu, Y. Liu, H. Yang, Q. Gong, K. Shi, L. Chen, Super-resolution fluorescence-assisted diffraction computational tomography reveals the three-dimensional landscape of the cellular organelle interactome. *Light Sci Appl* **9**, 11 (2020).

21. W.-H. Lee, Binary computer-generated holograms. *Appl. Optics* **18**, 3661–3669 (1979).

22. S. Shin, K. Kim, J. Yoon, Y. Park, Active illumination using a digital micromirror device for quantitative phase imaging. *Opt. Lett.* **40**, 5407–5410 (2015).

23. R. O. Chamgoulov, P. M. Lane, C. E. MacAulay, "Optical computed-tomography micoscope using digital spatial light modulation," in *SPIE Proceedings*, J.-A. Conchello, C. J. Cogswell, T. Wilson, Eds. (SPIE, 2004).

24. C. Kuang, Y. Ma, R. Zhou, J. Lee, G. Barbastathis, R. R. Dasari, Z. Yaqoob, P. T. C. So, Digital micromirror device-based laser-illumination Fourier ptychographic microscopy. *Opt. Express* **23**, 26999–27010 (2015).

25. S. Bianchi, F. Brasili, F. Saglimbeni, B. Cortese, R. Di Leonardo, Optical diffraction tomography of 3D microstructures using a low coherence source. *Opt. Express* **30**, 22321–22332 (2022).

26. K. Lee, K. Kim, G. Kim, S. Shin, Y. Park, Time-multiplexed structured illumination using a DMD for optical diffraction tomography. *Opt. Lett.* **42**, 999–1002 (2017).

27. S. Shin, D. Kim, K. Kim, Y. Park, Super-resolution three-dimensional fluorescence and optical diffraction tomography of live cells using structured illumination generated by a digital micromirror device. *Sci. Rep.* **8**, 9183 (2018).

28. D. Jin, R. Zhou, Z. Yaqoob, P. T. C. So, Dynamic spatial filtering using a digital micromirror device for high-speed optical diffraction tomography. *Opt. Express* **26**, 428–437 (2018).

29. S. K. Mirsky, I. Barnea, N. T. Shaked, Dynamic tomographic phase microscopy by double six-pack holography. *ACS Photonics* **9**, 1295–1303 (2022).

30. N. Zhou, J. Li, J. Sun, R. Zhang, Z. Bai, S. Zhou, Q. Chen, C. Zuo, Single-exposure 3D label-free microscopy based on color-multiplexed intensity diffraction tomography. *Opt. Lett.* **47**, 969–972 (2022).

31. H.-Y. Huang, Q.-Y. Yue, Y. Yang, R.-X. Wang, C.-S. Guo, Single-exposure multi-wavelength optical diffraction tomography based on space-angle dual multiplexing holography. *Opt. Lett.* **49**, 3066–3069 (2024).

32. P. T. Brown, R. Kruithoff, G. J. Seedorf, D. P. Shepherd, Multicolor structured illumination microscopy and quantitative control of polychromatic light with a digital micromirror device. *Biomed. Opt. Express* **12**, 3700–3716 (2021).

33. L. Tian, X. Li, K. Ramchandran, L. Waller, Multiplexed coded illumination for fourier ptychography with an LED array microscope. *Biomed. Opt. Express* **5**, 2376–2389 (2014).

34. A. Matlock, L. Tian, High-throughput, volumetric quantitative phase imaging with multiplexed intensity diffraction tomography. *Biomed. Opt. Express* **10**, 6432–6448 (2019).

35. J. Zhu, H. Wang, L. Tian, High-fidelity intensity diffraction tomography with a non-paraxial multiple-scattering model. *Opt. Express* **30**, 32808–32821 (2022).

36. S. Yang, J. Kim, M. E. Swartz, J. K. Eberhart, S. Chowdhury, DMD and microlens array as a switchable module for illumination angle scanning in optical diffraction tomography. *Biomed. Opt. Express* **15**, 5932–5946 (2024).

37. A. Beck, M. Teboulle, A fast iterative shrinkage-thresholding algorithm for linear inverse problems. *SIIMS* **2**, 183–202 (2009).

38. J. Lim, A. B. Ayoub, E. E. Antoine, D. Psaltis, High-fidelity optical diffraction tomography of multiple scattering samples. *Light Sci Appl* **8**, 82 (2019).

39. S. Kamdar, S. Shin, P. Leishangthem, L. F. Francis, X. Xu, X. Cheng, The colloidal nature of complex fluids enhances bacterial motility. *Nature* **603**, 819–823 (2022).

40. M. Kostakioti, M. Hadjifrangiskou, S. J. Hultgren, Bacterial biofilms: Development, dispersal, and therapeutic strategies in the dawn of the postantibiotic era. *Cold Spring Harb. Perspect. Med.* **3**, a010306–a010306 (2013).

41. P. Thibault, M. Dierolf, O. Bunk, A. Menzel, F. Pfeiffer, Probe retrieval in ptychographic coherent diffractive imaging. *Ultramicroscopy* **109**, 338–343 (2009).

42. H. Brenner, J. Happel, *Low Reynolds Number Hydrodynamics* (Springer Netherlands, 1983).

43. P. Holmqvist, J. K. G. Dhont, P. R. Lang, Colloidal dynamics near a wall studied by evanescent wave light scattering: Experimental and theoretical improvements and methodological limitations. *J. Chem. Phys.* **126**, 044707 (2007).

44. P. Sharma, S. Ghosh, S. Bhattacharya, A high-precision study of hindered diffusion near a wall. *Appl. Phys. Lett.* **97**, 104101 (2010).

45. S. Ferretti, S. Bianchi, G. Frangipane, R. Di Leonardo, A virtual reality interface for the immersive manipulation of live microscopic systems. *Sci. Rep.* **11**, 7610 (2021).

46. J. Wolfe, Structural analysis of basal bodies of the isolated oral apparatus of Tetrahymena pyriformis. *J. Cell Sci.* **6**, 679–700 (1970).

47. T. Fenchel, Respiration in heterotrophic unicellular eukaryotic organisms. *Protist* **165**, 485–492 (2014).

48. K. O'Holleran, M. Shaw, Optimized approaches for optical sectioning and resolution enhancement in 2D structured illumination microscopy. *Biomed. Opt. Express* **5**, 2580–2590 (2014).

49. D. Wloga, J. Frankel, From molecules to morphology: Cellular organization of *Tetrahymena thermophila*. *Methods Cell Biol.* **109**, 83–140 (2012).

50. A. W. J. Soh, C. G. Pearson, Ciliate cortical organization and dynamics for cell motility: Comparing ciliates and vertebrates. *J. Eukaryot. Microbiol.* **69**, e12880 (2022).

51. M. A. Fischler, R. C. Bolles, Random sample consensus: A paradigm for model fitting with applications to image analysis and automated cartography. *Commun. ACM* **24**, 381–395 (1981).

52. S. Chowdhury, W. J. Eldridge, A. Wax, J. Izatt, Refractive index tomography with structured illumination. *Optica* **4**, 537 (2017).

53. K. Wen, Z. Gao, R. Liu, X. Fang, Y. Ma, J. Zheng, S. An, T. Kozacki, P. Gao, Structured illumination phase and fluorescence microscopy for bioimaging. *Appl. Optics* **62**, 4871–4879 (2023).

54. I. E. Ivanov, E. Hirata-Miyasaki, T. Chandler, R. Cheloor-Kovilakam, Z. Liu, S. Pradeep, C. Liu, M. Bhave, S. Khadka, C. Arias, M. D. Leonetti, B. Huang, S. B. Mehta, Mantis: High-throughput 4D imaging and analysis of the molecular and physical architecture of cells. *PNAS Nexus* **3**, pgae323 (2024).

55. L.-H. Yeh, S. Chowdhury, N. A. Repina, L. Waller, Speckle-structured illumination for 3D phase and fluorescence computational microscopy. *Biomed. Opt. Express* **10**, 3635–3653 (2019).

56. Y. Mo, F. Feng, H. Mao, J. Fan, L. Chen, Structured illumination microscopy artefacts caused by illumination scattering. *Philos. Trans. R. Soc. London Ser. A Math. Phys. Eng. Sci.* **379**, 20200153 (2021).

57. M. Lisicki, M. F. Velho Rodrigues, R. E. Goldstein, E. Lauga, Swimming eukaryotic microorganisms exhibit a universal speed distribution. *Elife* **8**, e44907 (2019).

58. N. J. Brooks, C.-C. Liu, C.-L. Hsieh, Point spread function engineering for spiral phase interferometric scattering microscopy enables robust 3D single-particle tracking and characterization. *ACS Photonics* **11**, 5239–5250 (2024).

59. A. Matlock, J. Zhu, L. Tian, Multiple-scattering simulator-trained neural network for intensity diffraction tomography. *Opt. Express* **31**, 4094–4107 (2023).

60. Z. Wu, Y. Sun, A. Matlock, J. Liu, L. Tian, U. S. Kamilov, SIMBA: Scalable inversion in optical tomography using deep denoising priors. *IEEE J. Selected Topics Signal Process.* **14**, 1163–1175 (2020).

61. E. Bostan, R. Heckel, M. Chen, M. Kellman, L. Waller, Deep phase decoder: Self-calibrating phase microscopy with an untrained deep neural network. *Optica* **7**, 559 (2020).

62. R. Liu, Y. Sun, J. Zhu, L. Tian, U. S. Kamilov, Recovery of continuous 3D refractive index maps from discrete intensity-only measurements using neural fields. *Nat. Mach. Int.* **4**, 781–791 (2022).

63. R. Cao, F. L. Liu, L.-H. Yeh, L. Waller, "Dynamic structured illumination microscopy with a neural space-time model," in *2022 IEEE International Conference on Computational Photography (ICCP)* (IEEE, 2022), pp. 1–12.

64. D. Mahecic, W. L. Stepp, C. Zhang, J. Griffié, M. Weigert, S. Manley, Event-driven acquisition for content-enriched microscopy. *Nat. Methods* **19**, 1262–1267 (2022).

65. P. T. Brown, D. P. Shepherd, mcSIM v1.7.0 (2023); https://doi.org/10.5281/zenodo.14396655; https://github.com/QI2lab/mcSIM.

66. D. C. Ghiglia, L. A. Romero, Robust two-dimensional weighted and unweighted phase unwrapping that uses fast transforms and iterative methods. *J. Opt. Soc. Am. A* **11**, 107 (1994).

67. D. F. Galati, S. Bonney, Z. Kronenberg, C. Clarissa, M. Yandell, N. C. Elde, M. Jerka-Dziadosz, T. H. Giddings, J. Frankel, C. G. Pearson, DisAp-dependent striated fiber elongation is required to organize ciliary arrays. *J. Cell Biol.* **207**, 705–715 (2014).

68. W. Zhao, S. Zhao, L. Li, X. Huang, S. Xing, Y. Zhang, G. Qiu, Z. Han, Y. Shang, D. E. Sun, C. Shan, R. Wu, L. Gu, S. Zhang, R. Chen, J. Xiao, Y. Mo, J. Wang, W. Ji, X. Chen, B. Ding, Y. Liu, H. Mao, B. L. Song, J. Tan, J. Liu, H. Li, L. Chen, Sparse deconvolution improves the

resolution of live-cell super-resolution fluorescence microscopy. *Nat. Biotechnol.* **40**, 606–617 (2022).

69. M. Arzt, J. Deschamps, C. Schmied, T. Pietzsch, D. Schmidt, P. Tomancak, R. Haase, F. Jug, LABKIT: Labeling and segmentation toolkit for big image data. *Front. Comput. Sci.* **4**, 777728 (2022).

70. Q. Liu, R. He, C. Zhang, R. Zhang, J. Yuan, Bacterial surface swimming states revealed by TIRF microscopy. *Soft Matter* **20**, 661–671 (2024).

71. Y. C. Mazumdar, M. E. Smyser, J. D. Heyborne, M. N. Slipchenko, D. R. Guildenbecher, Megahertz-rate shock-wave distortion cancellation via phase conjugate digital in-line holography. *Nat. Commun.* **11**, 1129 (2020).

72. Z. Wang, R. Zhao, D. A. Wagenaar, D. Espino, L. Sheintuch, O. Benshlomo, W. Kang, C. Lee, W. Schmidt, A. Pammar, E. Zhu, J. Wang, G. C. L. Wong, R. Liang, P. Golshani, T. Hsiai, L. Gao, Kilohertz volumetric imaging of in-vivo dynamics using squeezed light field microscopy. bioRxiv 2024.03.23.586416 [Preprint] (2024). https://doi.org/10.1101/2024.03.23.586416.

73. R. Guo, Q. Yang, A. S. Chang, G. Hu, J. Greene, C. V. Gabel, S. You, L. Tian, EventLFM: Event camera integrated Fourier light field microscopy for ultrafast 3D imaging. *Light Sci Appl* **13**, 144 (2024).

74. D. R. Smith, D. G. Winters, R. A. Bartels, Submillisecond second harmonic holographic imaging of biological specimens in three dimensions. *Proc. Natl. Acad. Sci. U.S.A.* **110**, 18391–18396 (2013).

75. P. T. Brown, D. P. Shepherd, "Fourier synthesis of optical diffraction tomography patterns for kilohertz frame rate volumetric imaging," in *Biophotonics Congress: Optics in the Life Sciences 2023 (OMA, NTM, BODA, OMP, BRAIN)* (Optica Publishing Group, 2023), p. NTu1C.4.

76. P. T. Brown, D. P. Shepherd, "Multiplexing optical diffraction tomography patterns with Fourier synthesis for high-speed volumetric imaging," in *Quantitative Phase Imaging X*, Y. Park, Y. Liu, Eds. (SPIE, 2024), p. 22.

77. C. F. Bohren, D. R. Huffman, "Chapter 4. Absorption and scattering by a sphere" in *Absorption and Scattering of Light by Small Particles* (Wiley, 1983), pp. 82–129.

78. S. Prahl, miepython: Pure python implementation of Mie scattering v2.5.3 (2023); https://doi.org/10.5281/zenodo.8218010.

79. F. W. J. Olver, Error analysis of Miller's recurrence algorithm. *Math. Comput.* **18**, 65 (1964).

80. A. R. Barnett, "The calculation of spherical Bessel functions and Coulomb functions" in *Computational Atomic Physics: Electron and Positron Collisions with Atoms and Ions* (Springer Berlin Heidelberg, ed. 1, (1996), pp. 181–202.

81. P. T. Brown, D. P. Shepherd, Custom parts for mcSIM (2024); https://doi.org/10.5281/zenodo.10892403.

82. H.-W. Lu-Walther, M. Kielhorn, R. Förster, A. Jost, K. Wicker, R. Heintzmann, fastSIM: A practical implementation of fast structured illumination microscopy. *Methods Appl. Fluorescence* **3**, 014001 (2015).

83. F. Huang, T. M. Hartwich, F. E. Rivera-Molina, Y. Lin, W. C. Duim, J. J. Long, P. D. Uchil, J. R. Myers, M. A. Baird, W. Mothes, M. W. Davidson, D. Toomre, J. Bewersdorf, Video-rate nanoscopy using sCMOS camera-specific single-molecule localization algorithms. *Nat. Methods* **10**, 653–658 (2013).

84. J. Kostencka, T. Kozacki, Computational and experimental study on accuracy of off-axis reconstructions in optical diffraction tomography. *Optic. Eng.* **54**, 024107 (2015).

85. W. Krauze, A. Kuś, D. Śladowski, E. Skrzypek, M. Kujawińska, Reconstruction method for extended depth-of-field optical diffraction tomography. *Methods* **136**, 40–49 (2018).

86. A. J. Devaney, A filtered backpropagation algorithm for diffraction tomography. *Ultrason. Imaging* **4**, 336–350 (1982).

87. K. Kim, H. O. Yoon, M. Diez-Silva, M. Dao, R. R. Dasari, Y. K. Park, High-resolution three-dimensional imaging of red blood cells parasitized by Plasmodium falciparumandin situhemozoin crystals using optical diffraction tomography. *J. Biomed. Opt.* **19**, 011005 (2013).

88. K. Kim, K. S. Kim, H. Park, J. C. Ye, Y. Park, Real-time visualization of 3-D dynamic microscopic objects using optical diffraction tomography. *Opt. Express* **21**, 32269–32278 (2013).

89. P. Müller, M. Schürmann, J. Guck, ODTbrain: A Python library for full-view, dense diffraction tomography. *BMC Bioinformatics* **16**, 367 (2015).

90. J. Kostencka, T. Kozacki, A. Kuś, B. Kemper, M. Kujawińska, Holographic tomography with scanning of illumination: Space-domain reconstruction for spatially invariant accuracy. *Biomed. Opt. Express* **7**, 4086–4101 (2016).

91. J. Kostencka, T. Kozacki, “Space-domain, filtered backpropagation algorithm for tomographic configuration with scanning of illumination,” in *SPIE Proceedings*, C. Gorecki, A. K. Asundi, W. Osten, Eds. (SPIE, 2016).

92. L. Tian, L. Waller, 3D intensity and phase imaging from light field measurements in an LED array microscope. *Optica* **2**, 104 (2015).

93. U. S. Kamilov, I. N. Papadopoulos, M. H. Shoreh, A. Goy, C. Vonesch, M. Unser, D. Psaltis, Learning approach to optical tomography. *Optica* **2**, 517–522 (2015).

94. U. S. Kamilov, I. N. Papadopoulos, M. H. Shoreh, A. Goy, C. Vonesch, M. Unser, D. Psaltis, Optical tomographic image reconstruction based on beam propagation and sparse regularization. *IEEE Trans. Comput. Imaging* **2**, 59–70 (2016).

95. J. Lim, A. Goy, M. H. Shoreh, M. Unser, D. Psaltis, Learning tomography assessed using mie theory. *Phys. Rev. Appl.* **9**, 034027 (2018).

96. S. Chowdhury, M. Chen, R. Eckert, D. Ren, F. Wu, N. Repina, L. Waller, High-resolution 3D refractive index microscopy of multiple-scattering samples from intensity images. *Optica* **6**, 1211–1219 (2019).

97. U. S. Kamilov, D. Liu, H. Mansour, P. T. Boufounos, A recursive born approach to nonlinear inverse scattering. *IEEE Signal Process. Lett.* **23**, 1052–1056 (2016).

98. M. Chen, D. Ren, H.-Y. Liu, S. Chowdhury, L. Waller, Multi-layer born multiple-scattering model for 3D phase microscopy. *Optica* **7**, 394 (2020).

99. M. Lee, H. Hugonnet, Y. Park, Inverse problem solver for multiple light scattering using modified Born series. *Optica* **9**, 177 (2022).

100. S. Moser, A. Jesacher, M. Ritsch-Marte, Efficient and accurate intensity diffraction tomography of multiple-scattering samples. *Opt. Express* **31**, 18274–18289 (2023).

101. E. Soubies, T.-A. Pham, M. Unser, Efficient inversion of multiple-scattering model for optical diffraction tomography. *Opt. Express* **25**, 21786–21800 (2017).

102. T.-A. Pham, E. Soubies, A. Goy, J. Lim, F. Soulez, D. Psaltis, M. Unser, Versatile reconstruction framework for diffraction tomography with intensity measurements and multiple scattering. *Opt. Express* **26**, 2749–2763 (2018).

103. H.-Y. Liu, D. Liu, H. Mansour, P. T. Boufounos, L. Waller, U. S. Kamilov, SEAGLE: Sparsity-driven image reconstruction under multiple scattering. *IEEE Trans. Comput. Imaging* **4**, 73–86 (2018).

104. A. Chambolle, An algorithm for total variation minimization and applications. *J. Math. Imaging Vision* **20**, 89–97 (2004).

105. S. V. Venkatakrishnan, C. A. Bouman, B. Wohlberg, "Plug-and-Play priors for model based reconstruction," in *2013 IEEE Global Conference on Signal and Information Processing* (IEEE, (2013), pp. 945–948.

106. L.-H. Yeh, J. Dong, J. Zhong, L. Tian, M. Chen, G. Tang, M. Soltanolkotabi, L. Waller, Experimental robustness of Fourier ptychography phase retrieval algorithms. *Opt. Express* **23**, 33214–33240 (2015).

107. M. Slaney, A. Kak, L. Larsen, Limitations of imaging with first-order diffraction tomography. *IEEE Trans. Microwave Theory Tech.* **32**, 860–874 (1984).

108. D. Schröder, J. Deschamps, A. Dasgupta, U. Matti, J. Ries, Cost-efficient open source laser engine for microscopy. *Biomed. Opt. Express* **11**, 609–623 (2020).
